# Agentic LLM Compilation of Adaptive Sampling Policies for Low-Power IoT Sensing

**DOI:** 10.3390/s26175519

**Published:** 2026-08-31

**Authors:** Naghmeh Boyouk, Sara Behnamian, Mahshad Koohi Habibi Dehkordi

**Affiliations:** 1 Kempten Business School, Kempten University of Applied Sciences, Bahnhofstraße 61, 87435 Kempten, Germany; naghmeh.boyouk@stud.hs-kempten.de; 2 Globe Institute, University of Copenhagen, Øster Voldgade 5–7, 1350 Copenhagen K, Denmark; 3 Department of Biology, Lund University, 223 62 Lund, Sweden; 4 Department of Computer Science, New Jersey Institute of Technology, Newark, NJ 07102, USA; mk985@njit.edu

**Keywords:** adaptive sampling, energy-efficient sensing, large language models, LLM agents, program synthesis, duty cycling, time-series reconstruction, edge computing, Internet of Things

## Abstract

Battery-powered sensors must sample sparingly to conserve energy while still reconstructing the signal faithfully between measurements, and the decision logic must fit microcontroller-class hardware. We use a large language model (LLM) as an offline policy compiler: given a natural-language task description and a compact rule grammar, it returns policies as a few numeric thresholds and integer periods, under 100 bytes. No weights are fitted and the model never sees the raw series, only eight summary statistics: the approach is training-free but statistics-informed. Policies come from an agentic loop: a generator LLM proposes candidates, a deterministic evaluator scores each exactly on the recorded trace, and an optimizer LLM refines them over eight rounds. We evaluate Claude Opus 4.8, Claude Fable 5, ChatGPT 5.4 and ChatGPT 5.5, five seeds each, on the Beijing PM_2.5_ series against classical baselines, a budget-matched random search, and NSGA-II and ParEGO over the identical grammar. The loop improves on zero-shot generation and random search (pooled Wilcoxon $p<10^{-5}$): within a 0.20 duty-cycle budget the best model’s mean across five seeds reaches an RMSE of 21.79 against 25.09 for random search, and 15.98 against 20.57 within 0.30. The gain narrows at the tightest subsampling, which an interpolation-error bound explains through the signal’s curvature.

## 1. Introduction

Battery-powered sensors underpin environmental monitoring, industrial telemetry, and the wider Internet of Things (IoT) [1,2,3], yet their usefulness is bounded by a finite energy budget in which acquisition and communication are the dominant costs [4]. Because each measurement draws power, the rate at which a device samples is the primary lever on its operating lifetime: fewer samples mean longer sleep intervals and lower energy consumption. This creates a direct tension between energy and information, since sampling too sparsely leaves the underlying signal poorly reconstructed between observations. Deciding when to sample is therefore a first-order design problem for constrained sensing.

Adaptive sampling [5] attacks this tension by varying the acquisition rate with the signal itself, spending the budget where the signal is volatile and conserving it where the signal is quiescent [6]. In principle this improves fidelity at a fixed energy cost, but two practical obstacles remain. First, classical adaptive and event-driven controllers expose sensitivity parameters that must be re-tuned for each deployment [7]. Second, any decision logic must ultimately execute on microcontroller-class hardware; while the TinyML movement has made on-device inference feasible [8,9], a learned model still carries a memory footprint, a per-inference cost, and a requirement to be fitted to the target signal before deployment. What is deployed on the device must be cheap, and ideally interpretable, however sophisticated the process that produced it.

That separation is the core of our approach: the intelligence used to construct a sampling policy may be arbitrarily expensive and entirely offline, whereas the artefact that runs on the device must be trivially cheap. Concretely, we use a large language model (LLM) [10,11,12] as an offline policy compiler. Given a natural-language description of the sensing task and a compact rule grammar, the model returns candidate policies expressed directly in that grammar—a few numeric thresholds and integer periods, at most 92 bytes of data alongside a fixed 296-byte interpreter (Section 4.8)—which are then deployed with no further model involvement and hence zero on-device LLM inference cost. This is motivated by the rapid progress of LLMs from few-shot predictors [13] to systems that emit executable code [14] and discover competitive heuristics through program search or optimisation [15,16].

An LLM that emits plausible-looking policies is not, by itself, evidence of value. The decisive question is whether the model confers any advantage beyond unguided exploration of the same policy space. We therefore embed the model in an agentic optimization loop—a generator LLM proposes policies, a deterministic evaluator scores each exactly on the recorded trace, and an optimizer LLM refines them from that feedback over several rounds—and evaluate the resulting policies under a trace-driven protocol on a real environmental signal, the Beijing PM_2.5_ series [17], against classical fixed-rate, delta-triggered adaptive-period, and prediction-based baselines. The key control is a random search over the identical grammar, using at least as many policy evaluations as the LLM loop, so that any advantage is not simply due to trying more candidate policies. Random search alone, however, is a weak reference for a discrete design space of this kind, where evolutionary and surrogate-based optimisers are the standard tools. We therefore also compare against NSGA-II [18] and ParEGO [19] over the identical grammar, scored by the identical evaluator (Section 4.7), so that the question is not whether guidance beats no guidance but whether it beats competent guidance at a matched evaluation budget. Across four contemporary LLMs and five seeds each, the feedback loop expands the zero-shot policy archive and improves over unguided random search under this policy-evaluation control (pooled Wilcoxon $p<10^{-5}$ in both cases), surpassing random search in best-attainable fidelity across most of the operating range. We complement the empirical study with an analytical characterisation that explains where and why the gain is largest, and why it narrows at the most aggressive subsampling. The scope of that evidence should be stated at the outset: policies are compiled on a single series. Section 4.6 replays every compiled policy unchanged on four further channels recorded synchronously at the same site, and finds the margin over unguided search tracks the signal’s curvature as the analysis predicts—but it transfers the compiled artefacts rather than re-running the loop, and all five channels share one site and one sampling rate. Section 4.10 closes that gap in the other direction, re-running the loop under a chronological split of the generating series and measuring how much of the margin survives on a held-out year. Section 5.1 states what remains untested.

The contributions of this paper are as follows:

- We formulate LLM-as-compiler for adaptive sampling: an offline model translates a natural-language task specification into compact, interpretable rule-based policies that are immediately deployable on microcontroller-class hardware at negligible cost.
- We cast policy generation as an offline agentic loop (generator–evaluator–optimizer) and provide a rigorous, trace-driven benchmark on a real environmental signal that compares four contemporary LLMs, across five seeds each, against classical baselines and, crucially, against a budget-matched random search over the same grammar, so that any advantage is attributable to guidance rather than to exploration alone, and against NSGA-II and ParEGO over the same grammar at matched evaluation budget.
- We give an analytical characterisation of the grammar, including an exact pointwise interpolation-error bound and a grammar-wide worst-case guarantee, which link reconstruction error to the signal’s curvature and thereby clarify what the grammar’s first-order features can and cannot achieve.
- We report a statistically supported result: the feedback loop expands the zero-shot policy archive and improves over budget-matched random search, and surpasses random search in best-attainable fidelity across most of the duty-cycle range, while the resulting policies remain compact, interpretable, and free of any on-device model. The gain is budget-dependent, vanishing only at the tightest subsampling, which we explain analytically. The result is established on policies compiled from one series; Section 4.6 measures how far the compiled artefacts transfer, and Section 5.1 states what remains untested.

The remainder of the paper is organised as follows. Section 2 reviews the related work across energy-efficient sensing, event-driven sampling, on-device intelligence, black-box optimisation over structured design spaces, and LLM-based program generation, and positions our contribution against it. Section 3 formalises the problem, the reconstruction and duty-cycle model, the policy grammar, the LLM-based generation procedure, the baselines, the evaluation metrics, and the analytical properties of the grammar. Section 4 reports the empirical comparison on the Beijing PM_2.5_ series and its extension to four further sensor channels. Section 5 discusses what the results do and do not establish, states the limitations of the study, and outlines future work. Section 6 concludes.

## 2. Literature Review

The problem of sampling a signal sparsely while preserving reconstruction fidelity sits at the intersection of several bodies of work: energy-efficient sensing, event-driven sampling and reconstruction, on-device machine learning, black-box optimisation over structured design spaces, and—most recently—the use of large language models (LLMs) to synthesise and iteratively refine executable artefacts. We review each in turn and position the present contribution against them.

### 2.1. Energy-Efficient and Adaptive Sensing

Prolonging the lifetime of battery-powered sensors is a long-standing concern in wireless sensor networks, where communication and acquisition dominate the energy budget and duty cycling is the principal lever for saving power [4]. Fixed-rate (periodic) sampling is the simplest strategy and provides the baseline duty-cycle/fidelity tradeoff, but it spends the same budget on quiescent and volatile stretches alike. Adaptive sampling addresses this by modulating the acquisition rate in response to observed signal dynamics, sampling densely when the signal is changing and sparsely otherwise [6]. Such schemes reduce energy for a given fidelity, but they typically expose sensitivity parameters that must be tuned per deployment, and the resulting controllers are rarely characterised across the full energy–fidelity frontier—which is the frontier-level, tuning-free comparison we pursue here.

### 2.2. Event-Driven Sampling and Reconstruction

A complementary line replaces clock-driven acquisition with event-driven reporting. The idea has deep roots in control theory, where Lebesgue (event-based) sampling has long been contrasted with periodic Riemann sampling and shown to achieve comparable performance at a lower average rate [20], and where event-triggered and self-triggered control formalise when a fresh sample or update is genuinely needed [21]. In the sensing literature the classical delta-triggered adaptive-period scheme instantiates this principle directly, transmitting a new value only when the signal has changed by more than a fixed threshold since the last report, thereby concentrating communication where information content is highest [7]. Whatever the sampling rule, the values between samples must be reconstructed. Model-free piecewise-linear interpolation is attractive on constrained hardware because it carries no state and can be reproduced by a downstream collector without additional assumptions, which is the operator we adopt. More expressive reconstructions are possible; variational and total-variation-regularised methods recover signals by penalising non-smoothness and can outperform interpolation when a richer prior is warranted [22], and the same regulariser has been embedded in unsupervised neural architectures for related dense estimation problems [23]; spline-based reconstructions [24] are a further option, at the cost of solving an optimisation problem the sensor itself cannot afford. Compressed sensing is the other major route to sub-Nyquist acquisition, recovering a signal from far fewer measurements than the Nyquist rate when it is sparse in some basis [25,26], and it has been applied to sensor networks through compressive data gathering [27]. We exclude it deliberately, for three reasons. It acquires randomised linear projections of the signal rather than choosing when to sample, which is a different acquisition model from duty-cycled scheduling and not expressible in a causal per-sample rule. Its decoder solves a convex optimisation problem, so the frugality the present work is organised around would be moved off the sensor rather than achieved. And its guarantees rest on sparsity in a known basis, which the PM_2.5_ series is not shown to satisfy. Compressive acquisition is therefore a complementary design point rather than a baseline here.

### 2.3. On-Device Intelligence and TinyML

The TinyML movement pushes machine-learning inference onto microcontroller-class devices, supported by dedicated runtimes and benchmarks [8,9]. These advances make learned, adaptive behaviour deployable in principle, but a trained model still carries a memory footprint and per-inference cost, and it must be fitted to the target signal before deployment. This motivates our design: rather than deploying a learned model, we deploy a handful of numeric thresholds and confine all expensive computation offline.

### 2.4. Black-Box Optimisation over Structured Design Spaces

Choosing a rule set from a grammar is a black-box optimisation problem over a structured, partly discrete space, and two families dominate that setting. Evolutionary multi-objective algorithms approximate a whole Pareto front in one run; NSGA-II is the standard reference, combining fast non-dominated sorting with crowding-distance selection to maintain diversity [18]. Surrogate-based methods instead fit a probabilistic model of the objective and spend evaluations where the model expects improvement, which matters when each evaluation is expensive; ParEGO extends this to the multi-objective case by scalarising with a fresh random weight vector at each iteration and optimising expected improvement over a Gaussian-process surrogate [19]. Grammar-aware variants search the derivation structure directly rather than a flat encoding [28].

These methods are the natural point of comparison for the present work, and the relevant question is not whether they can solve the problem—given enough evaluations they can—but how many evaluations they need, and what has to be built before the first one can be run. Each requires an encoding, operators or a kernel, and a tuned configuration. We therefore treat them as controls rather than as related applications, implement both over the identical grammar with the identical evaluator, and report the budget at which each overtakes the loop (Section 4.7).

### 2.5. Large Language Models as Program Generators and Iterative Optimizers

LLMs are now applied across healthcare, the Internet of Things (IoT), robotics, software engineering and scientific discovery [29,30,31,32]. Beyond their role as conversational assistants, LLMs have progressed from few-shot predictors [13] to systems that emit executable code [14] and, more strikingly, that discover competitive algorithms and heuristics through program search [15] or act as black-box optimisers [16]. These results suggest LLMs can serve not only as end-user assistants but as compilers that translate a natural-language specification into a compact, executable artefact.

Beyond one-shot generation, a growing body of work places the model in a feedback loop, alternating generation with evaluation so that later outputs are conditioned on the measured quality of earlier ones. Self-Refine and Reflexion improve a model’s own outputs from verbal or scalar feedback across iterations [33,34]; Eureka evolves reward functions by repeatedly generating candidates, scoring them in simulation, and refining the best [35]; and EvoPrompt couples an LLM with evolutionary operators to optimise prompts against a fitness signal [36]. FunSearch likewise pairs generation with an automated evaluator to search a space of programs [15]. The common ingredient across these systems is an external, trustworthy signal that grounds the refinement. Our setting supplies exactly such a signal: because every candidate policy can be scored exactly on the recorded trace, the evaluator is deterministic ground truth rather than a model estimate. This both drives the loop and makes the central comparison—against a budget-matched random search over the same grammar—unambiguous, since any advantage the loop shows cannot be an artefact of a noisy or model-based score.

The present authors have previously applied LLMs to structured, evidence-grounded classification tasks [37,38]. Those studies concern a different problem and are cited only for the narrow point they support: that a natural-language specification plus a constrained output format yields reliably well-formed outputs, which is the property the compiler view depends on. The present paper takes that view to its conclusion for adaptive sensing: an LLM is used offline, within a feedback-driven optimization loop, to compile sampling policies expressed directly in a constrained grammar, incurring zero on-device LLM inference cost.

Table 1 summarises the comparison.

Across these lines of work, adaptivity, on-device frugality, and interpretability are each achievable in isolation, but rarely together and never, to our knowledge, by having an LLM compile compact, human-readable sampling policies through an offline generate–evaluate–improve loop and evaluating them head-to-head against classical baselines, a budget-matched random search, and standard black-box optimisers over the identical policy grammar. Closing that gap—and, in particular, establishing whether the guided procedure confers an advantage beyond unguided exploration of the same space under a controlled policy-evaluation budget—is the aim of the present study.

## 3. Materials and Methods

**Figure 1:**
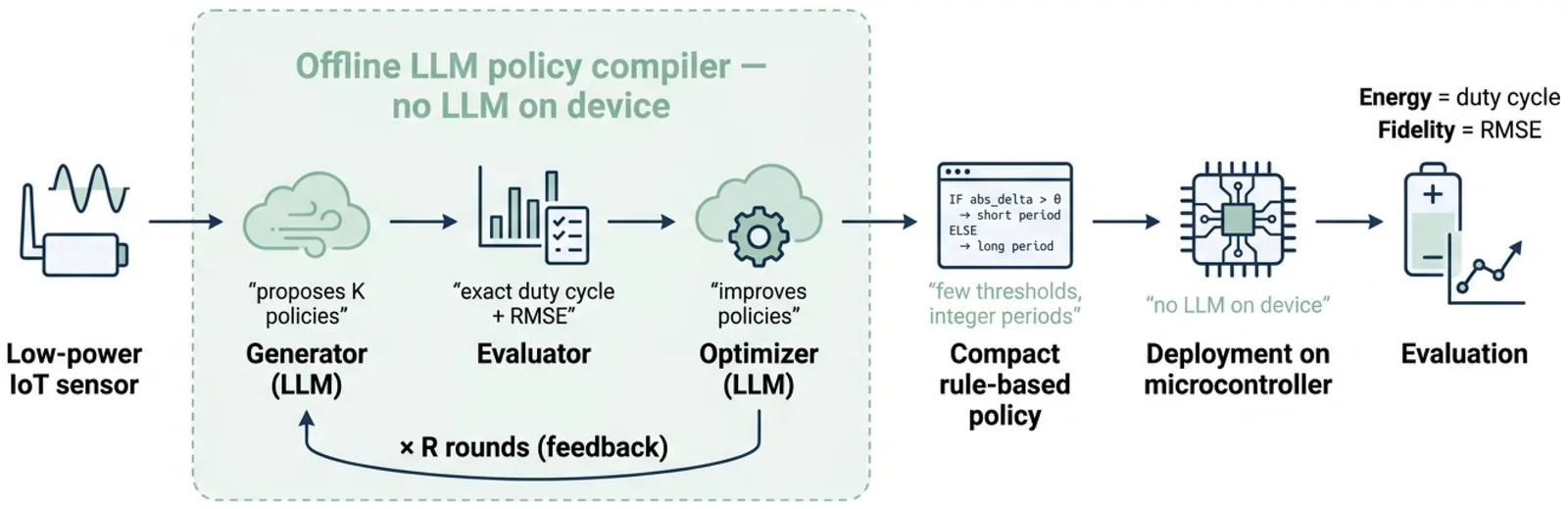
Graphical overview of the proposed offline LLM-as-policy-compiler framework. Policies are produced entirely off-device by an iterative agentic loop: a generator LLM proposes candidate policies, a deterministic evaluator scores each on the recorded trace (duty cycle and RMSE), and an optimizer LLM refines them from that feedback, repeating for *R* rounds. All LLM involvement occurs offline through provider APIs; the deployed artefact contains only simple threshold-based rules and integer sampling periods, so no LLM is executed on the microcontroller. Policies are evaluated using duty cycle as an energy proxy and RMSE as a reconstruction fidelity measure.

Figure 1 summarises the overall offline policy-generation and trace-driven evaluation workflow. The following subsections define each component of this workflow in detail.

### 3.1. Problem Formulation

We consider a battery-powered sensor that observes a scalar physical quantity over time. Let $x=(x_{1},x_{2},\ldots ,x_{N})$ denote the fully sampled reference signal of length *N*. A sampling policy π selects a subset of time indices $S_{\pi }=\left\{t_{1},t_{2},\ldots ,t_{|S_{\pi }|}\right\}\subseteq \left\{1,\ldots ,N\right\}$ at which the device actually acquires a measurement. Between sampled instants the signal is not observed and must be reconstructed. We denote the reconstruction obtained from the sampled points by $\hat{x}=R\left(x,S_{\pi }\right)$.

We evaluate a policy along two competing axes. The first is an energy proxy, the duty cycle, defined as the fraction of time steps at which the device samples,

(1) $$d\left(\pi \right)\;=\;\frac{|S_{\pi }|}{N},$$

which is monotonically related to the sensing energy budget: fewer samples imply longer sleep intervals and lower energy consumption. The second is reconstruction fidelity, measured as the root-mean-square error between the reconstruction and the reference signal over all *N* time steps,

(2) $$\mathrm{RMSE}\left(\pi \right)\;=\;\sqrt{\frac{1}{N}\sum_{t=1}^{N}{\left({\hat{x}}_{t}-x_{t}\right)}^{2}}.$$

The design objective trades two competing quantities, and we state it as a bi-objective problem,

(3) $$\min_{\pi }\;\left(d(\pi ),\;\mathrm{RMSE}(\pi )\right),$$

whose solution is not a single policy but the set of Pareto-optimal ones. This is the form the evaluation measures, through the attained front and its hypervolume (Section 3.7).

A single point on that front is picked out by the ε-constraint scalarisation of Equation (3),

(4) $$\min_{\pi }\;d\left(\pi \right)\;\mathrm{subject}\;\mathrm{to}\;\mathrm{RMSE}\left(\pi \right)\leq \varepsilon ,$$

which is the form a deployment with a fixed error tolerance ε would solve. Sweeping ε traces the front of Equation (3), so the two are the same problem stated at different granularity. We give both because the constrained form is what a practitioner faces and the bi-objective form is what can be evaluated without committing to one tolerance. The budget-wise results of Section 4.4 are the same constraint applied to the other objective: *d* is fixed and the best attainable RMSE is reported.

Because the choice of $S_{\pi }$ is a combinatorial selection over $2^{N}$ possible subsets, exact optimisation of either form is intractable at the scales considered here, which motivates the use of compact, causal heuristic policies.

### 3.2. Causal Reconstruction and Duty-Cycle Model

Reconstruction is performed by piecewise-linear interpolation between consecutive sampled values: for any *t* with $t_{i}\leq t\leq t_{i+1}$ and $t_{i},t_{i+1}\in S_{\pi }$,

(5) $${\hat{x}}_{t}\;=\;x_{t_{i}}+\left(x_{t_{i+1}}-x_{t_{i}}\right)\;\frac{t-t_{i}}{t_{i+1}-t_{i}}.$$

We use interpolation because it requires no model state and can be reproduced by a downstream collector without further assumptions. The first index is always sampled ($t_{1}=1$) to initialise the reconstruction. If the final scheduled sample occurs before the end of the trace, the remaining tail is reconstructed by holding the last sampled value constant. This trailing zero-order-hold segment is included in the RMSE calculation but is not covered by the interpolation bound of Proposition 1, which applies only to indices bracketed by two samples.

The energy proxy in Equation (1) counts sampling events. This abstracts away the absolute per-sample energy cost $e_{s}$ and any radio cost; the total sensing energy of a policy is $E\left(\pi \right)=\;|S_{\pi }|\;e_{s}$, so duty cycle and energy are proportional and the tradeoff curves are invariant to the constant $e_{s}$. We therefore report duty cycle throughout as a hardware-independent energy surrogate.

### 3.3. Policy Grammar

A policy is represented as an ordered list of conditional rules together with a fallback period. At each sampling instant the policy is consulted to decide the waiting period $p\in \{1,\ldots ,p_{\max}\}$ (in time steps) until the next sample, with $p_{\max}=48$. Rules are evaluated in order and the first whose condition is satisfied determines *p*; if no rule matches, a default period $p_{\mathrm{def}}$ is used. The resulting period is clamped to $\left[1,p_{\max}\right]$. The cap $p_{\max}=48$ corresponds to two days at the hourly sampling rate of the series and admits duty cycles as low as 1/48≈0.021, roughly five times below the tightest budget reported in Section 4.4. It is also the parameter that controls the worst-case guarantee of Proposition 1, which degrades as $p_{\max}^{2}$, so a larger cap buys access to sparser policies at the cost of a weaker structural bound. Both $p_{\max}$ and the smoothing factor α introduced below were fixed a priori; Section 4.9 reports how the results respond to each.

**Figure 2:**
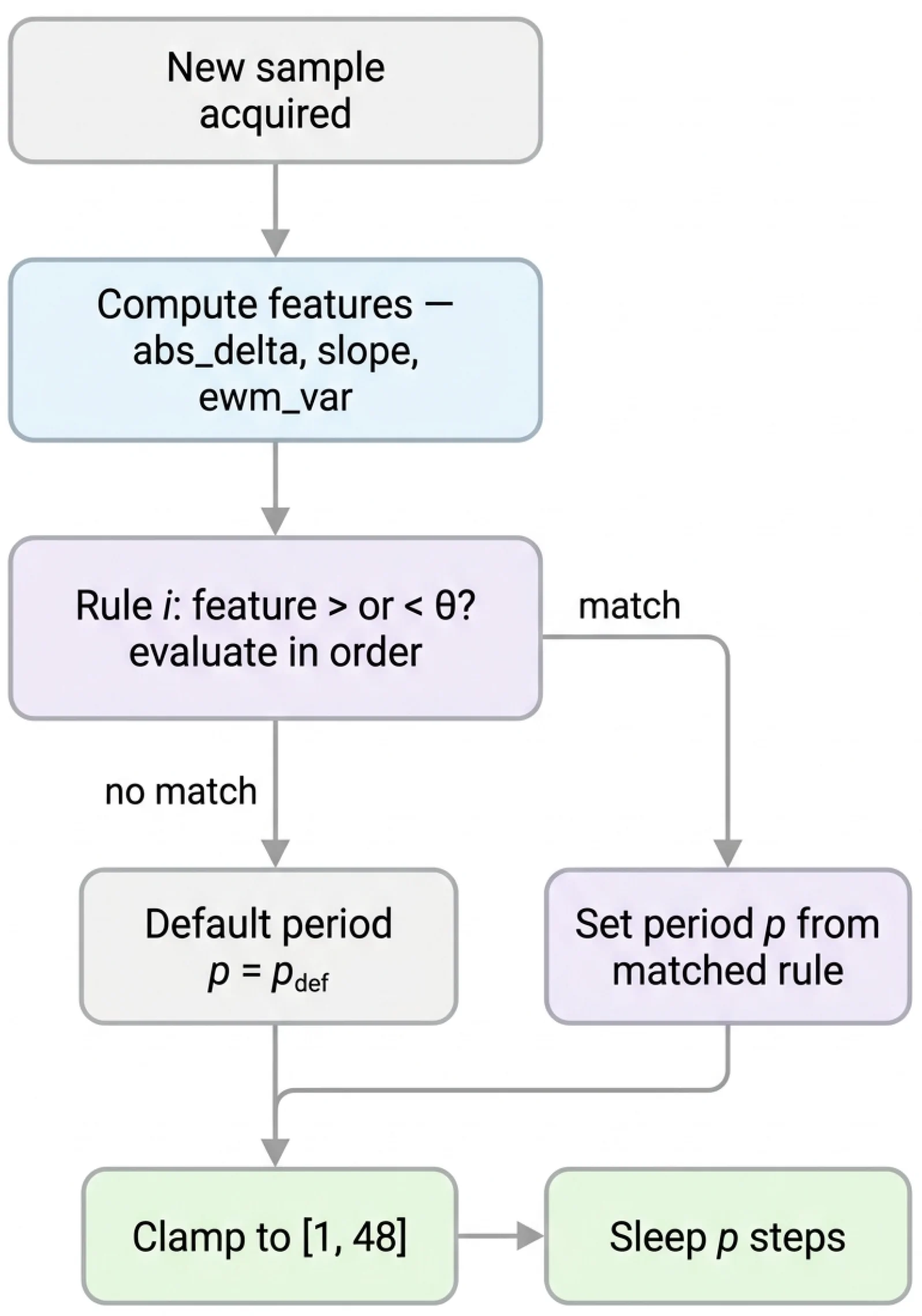
Decision logic of the compact rule-based sampling grammar. After each new sample is acquired, causal features are computed from already observed values. Rules are evaluated in order; the first matching rule sets the next sampling period, while the default period is used if no rule matches. The period is then clamped to the allowed range before the sensor sleeps until the next sample. The notation [1,48] denotes the allowed range of integer sampling periods. Gray indicates acquisition or default actions, blue indicates feature computation, purple indicates rule evaluation and period selection, and green indicates period clamping and sleep.

Crucially, every rule condition is a single threshold test on a feature that is computable in O(1) time and memory from the values the device has already sampled; the policy never accesses unsampled data. Let $x_{t_{i}}$ be the most recent sample and $x_{t_{i-1}}$ the one before it. The available features are

(6) $$\begin{array}{cc} \mathrm{abs}\_\mathrm{delta} & =\left|x_{t_{i}}-x_{t_{i-1}}\right|, \end{array}$$

(7) $$\begin{array}{cc} \mathrm{rel}\_\mathrm{delta} & =\frac{\left|x_{t_{i}}-x_{t_{i-1}}\right|}{\left|x_{t_{i-1}}\right|+1}, \end{array}$$

(8) $$\begin{array}{cc} \mathrm{slope} & =\frac{x_{t_{i}}-x_{t_{i-1}}}{t_{i}-t_{i-1}}, \end{array}$$

(9) $$\begin{array}{cc} \mathrm{time}\_\mathrm{since} & =0, \end{array}$$

where time_since is retained for grammar completeness; in the present one-step decision formulation it is zero at every decision point (the period is chosen before any waiting elapses) and therefore acts only as a constant feature. The exponentially weighted variance ewm_var of the sampled values is maintained incrementally with smoothing factor α=0.2: on acquiring a new sample $x_{t_{i}}$,

(10) $$\begin{array}{cc} \delta & =x_{t_{i}}-\mu , \end{array}$$

(11) $$\begin{array}{cc} \mu & \leftarrow \mu +\alpha \;\delta , \end{array}$$

(12) $$\begin{array}{cc} v & \leftarrow \left(1-\alpha \right)\left(v+\alpha \;\delta^{2}\right), \end{array}$$

where μ and *v* are the running mean and variance estimates and ewm_var =v. A rule condition compares a feature *f* against a threshold θ using either f>θ or f<θ. The smoothing factor α=0.2 was likewise fixed in advance rather than tuned; its effect is quantified in Section 4.9. Figure 2 illustrates this rule-evaluation process.

The entire policy is thus a small set of numeric thresholds and integer periods: at most 92 bytes under the encoding of Section 4.8, evaluated with a constant number of comparisons at each decision point.

### 3.4. Dataset

We evaluate on the Beijing PM_2.5_ air-quality dataset from the UCI Machine Learning Repository [17], comprising hourly fine-particulate-matter concentration readings recorded over 2010–2014. After forward/backward filling of missing entries the series contains N=43,824 hourly observations. The signal has mean 98.3, standard deviation 90.9, range [0,994], lag-1 autocorrelation 0.966, and lag-24 autocorrelation 0.402; the median hourly absolute change is 7.0 and the 95th percentile is 42.0. These summary statistics indicate a signal that is strongly correlated at short lags but subject to occasional large excursions, which is the regime in which adaptive sampling is expected to help.

We adopt a trace-driven evaluation: each policy is replayed over the recorded series, and its samples, reconstruction, duty cycle, and RMSE are computed offline according to Equations (1)–(5). This measures the sampling behaviour a policy would have produced on the recorded signal; validation on live battery-powered hardware is left to future work.

The main experiments of Section 4.1, Section 4.2, Section 4.3, Section 4.4, Section 4.5, Section 4.6, Section 4.7, Section 4.8 and Section 4.9 use no train/test partition: every policy, from every method, is scored on the whole series. This keeps the comparison between methods exact, since all of them see identical data, but it is an in-sample evaluation, and we state that plainly rather than leave it to be inferred. The thresholds a compiled policy carries were shaped by scores measured on the same series it is then reported on. Two things bound how far this can flatter the result: the policies are of very low capacity, a median of five rules and at most eleven (Section 4.8), so there is little room to fit a 43,824-point series; and Section 4.6 replays every compiled policy unchanged on four further channels, which tests the artefacts out of sample even though it is not a held-out segment of the generating signal.

Because that bound is an argument rather than a measurement, Section 4.10 additionally reports a chronological split: the loop is re-run compiling on 2010–2013 and scoring on the held-out 2014 segment, with the prompt statistics, the evaluator and the controls all restricted to the training segment. That experiment measures directly how much of the reported advantage is trace-specific fitting.

### 3.5. LLM-Based Policy Generation via an Agentic Optimization Loop

Policies are produced by an iterative agentic loop in which the model repeatedly proposes policies, receives their exact measured scores, and proposes improved ones. The loop comprises three roles.

Generator (LLM).

Given the sensing objective, the signal summary statistics of Section 3.4, the features of Equations (6)–(12), and the JSON grammar with one worked example, the model returns a batch of *K* candidate policies (K=12).

The prompt carries eight scalars—mean, standard deviation, minimum, maximum, lag-1 and lag-24 autocorrelation, median absolute change, and its 95th percentile—and no raw samples. The method is therefore statistics-informed, not signal-agnostic, and we describe it that way throughout. It remains training-free in the strict sense that no weights are fitted and the model never sees the series, but it is not oblivious to the dataset, and a deployment on a new signal would require the same eight numbers.

Evaluator (deterministic).

Each candidate is scored exactly by the trace-driven harness of Section 3.2, yielding its (d,RMSE). The evaluator is deliberately not an LLM: the score is ground truth computed from the recorded series, and an LLM estimate would be unreliable and would compromise the comparison. The evaluator also compiles a compact feedback digest—the current non-dominated policies with their measured scores, and the duty-cycle budgets at which a classical baseline currently attains lower error.

Optimizer (LLM).

The digest is returned to the model, which is asked to propose *K* improved policies that reduce RMSE in the budgets where it is currently behind without exceeding the corresponding duty budget; it may refine structures from the current front.

These roles are iterated for *R* rounds (R=8). Round 0 is a pure zero-shot generation with no feedback and is retained as an explicit baseline—the zero-shot condition against which the loop’s gain is measured. After each round the newly generated policies are pooled with all previous ones and the running non-dominated (Pareto) front is recomputed; the loop terminates after *R* rounds or when the front’s hypervolume (Section 3.7) has not improved for three consecutive rounds. A single run therefore yields up to (R+1)×K policies per model. Both *K* and *R* were fixed in advance rather than tuned. Because *R* enters the loop only through the termination condition, a run configured with R=r ends in exactly the state a run configured with R=8 occupies after its *r*-th round; Section 4.1 uses this to report an exact ablation over R=0,…,8, together with the marginal contribution of each proposal, which bounds what a larger *K* could have added.

To probe sensitivity to prompt wording we define four prompt variants: the base prompt; a variant emphasising the low-power regime; a variant requesting explicit step-by-step reasoning about signal curvature before answering; and a variant that asks for policies each targeting a distinct duty-cycle band. Each run uses one variant, selected by the run seed.

We evaluate four contemporary models—Claude Opus 4.8, Claude Fable 5, ChatGPT 5.4, and ChatGPT 5.5—each accessed through its provider API (not the chat interface) in July 2026, with five independent seeds per model (n=5). We request temperature 0; the GPT models reject a temperature of 0 and the Claude models do not accept a temperature parameter at all, so each model runs at its provider default. The iso-budget arms of Section 4.2, together with a contemporaneous (8,12) replication, were generated in August 2026 on two of the four models under the identical procedure and code. Beyond selecting the prompt variant, the seed permutes the subset of scored policies shown in the feedback digest and the seed of the random-search control, so repeated runs follow genuinely different trajectories. The generation step is therefore not reproducible: at each provider’s default temperature, an identical prompt may return different policies. What is deterministic is everything downstream of the returned policies—the evaluator, the front construction and the hypervolume are exact functions of the policy set, so every number in this paper is reproducible from the released policies. The variability that remains is in the generator, and it is what the five seeds per model are there to measure (Section 4.1). Representative compiled policies are given in Appendix A; both prompt templates and all four variants are reproduced in full in Appendix B, and the complete archive of all generated policies with their exact scores is released in the repository named in the data availability statement.

### 3.6. Baselines

We compare the LLM-generated policies against four families of baselines, all evaluated under the identical trace-driven protocol and, where applicable, expressed in the same policy grammar so that comparisons are made on equal terms.

Fixed-rate sampling.

A single constant period p∈{1,2,3,4,6,8,12,16,24,36,48}, giving a reference curve of the naive duty-cycle/fidelity tradeoff.

Delta-triggered adaptive-period baseline.

We include a simple event-inspired baseline in the same executable grammar: if the most recent sample-to-sample change exceeds a threshold δ∈{2,5,10,20,40,80}, the next sampling period is set to one step; otherwise a default period of 12 steps is used. This is not a full communication-level send-on-delta protocol, but a grammar-compatible delta-triggered sampling rule.

Prediction-based adaptive sampling.

A short period is used while the signal is changing rapidly (large slope or ewm_var) and a long period otherwise, with sensitivity τ∈{2,5,10,20,40}, representing standard adaptive-sampling heuristics.

Random search over the grammar.

We draw policies uniformly at random from the same grammar (one to three rules per policy; random features, operators, thresholds, and periods) under a fixed per-seed random seed. To keep the comparison conservative, the number of random policies is set to at least the total number of LLM-generated policies in the corresponding run, with a minimum of 300 random policies. This is the key control: it isolates whether the LLM confers an advantage beyond unguided exploration of the identical policy space under a controlled policy-evaluation budget. Any benefit attributable to the LLM must manifest as policies that dominate the random-search Pareto front at comparable duty cycles.

### 3.7. Metrics and Comparison

Each policy is summarised by the pair (d,RMSE) from Equations (1) and (2). A policy $\pi_{a}$ Pareto-dominates $\pi_{b}$ if $d\left(\pi_{a}\right)\leq d\left(\pi_{b}\right)$ and $\mathrm{RMSE}\left(\pi_{a}\right)\leq \mathrm{RMSE}\left(\pi_{b}\right)$ with at least one inequality strict. For each method and run we report the non-dominated (Pareto) front of its attained (d,RMSE) points.

To summarise a whole front by a single number we use its hypervolume indicator [39]: the area of the (d,RMSE) region dominated by the front, measured toward the origin from a fixed reference point $\left(d_{\mathrm{ref}},{\mathrm{RMSE}}_{\mathrm{ref}}\right)=\left(1.0,100\right)$. A larger hypervolume indicates a front that is simultaneously lower in error and lower in duty cycle, and the same reference point is used for every method and run so the values are directly comparable. We additionally report, at duty-cycle budgets d≤{0.10,0.15,0.20,0.30,0.50}, the best (lowest) RMSE attained within each budget.

Because LLM outputs vary between runs, all quantities are computed over the five seeds per model and reported with dispersion. We summarise each model’s loop hypervolume by its median across seeds with a bootstrap 95% confidence interval, and test the loop against two references using the paired Wilcoxon signed-rank test across seeds: (i) the round-0 zero-shot front, and (ii) the budget-matched random search. To assess whether models differ from one another we apply a Friedman omnibus test across models. Friedman rather than Kruskal–Wallis is used because the design is paired: a given seed selects the same prompt variant for every model, so the five observations per model are matched rather than independent. We report the omnibus result only, and deliberately do not follow it with pairwise tests: at n=5 the paired Wilcoxon cannot reach p<0.05, so pairwise *p*-values would be uninformative about ordering. Budget-wise best-RMSE values are reported as the mean across seeds with a 95% confidence interval. All methods are evaluated on the same signal with the same reconstruction operator, so reported differences reflect the sampling decisions alone.

### 3.8. Analytical Properties of the Grammar

The reconstruction operator of Section 3.2 admits an exact error characterisation that clarifies what any policy in our grammar can and cannot achieve. Let $e_{t}={\hat{x}}_{t}-x_{t}$ denote the reconstruction error and let ${\left(\Delta^{2}x\right)}_{s}=x_{s+1}-2x_{s}+x_{s-1}$ be the second difference (discrete curvature) of the signal.

**Lemma** **1** (Pointwise interpolation error). *Let $t_{i},t_{i+1}\in S_{\pi }$ be consecutive samples bracketing a gap of length $g=t_{i+1}-t_{i}$. For g≥2, let $M=\max_{t_{i}<s<t_{i+1}}\left|{\left(\Delta^{2}x\right)}_{s}\right|$; for g=1 set M=0. Then for every interior point $t_{i}+j$ with 0≤j≤g,*

(13) $$|e_{t_{i}+j}|\;\leq \;M\;\frac{j\;(g-j)}{2}\;\leq \;\frac{M\;g^{2}}{8}.$$

**Proof.**  Reindex the gap locally by j=0,…,g, so that position $t_{i}+j$ carries error $e_{j}$, with $e_{0}=e_{g}=0$ because interpolation is exact at the samples. As the interpolant (5) is affine in *j*, its second difference vanishes on the interior, so ${\left(\Delta^{2}e\right)}_{j}=-{\left(\Delta^{2}x\right)}_{t_{i}+j}=:-b_{j}$ with $|b_{j}|\leq M$ for 0<j<g. Hence *e* solves the discrete Dirichlet problem $-{\left(\Delta^{2}e\right)}_{j}=b_{j}$, $e_{0}=e_{g}=0$, whose solution is $e_{j}=\sum_{k=1}^{g-1}G\left(j,k\right)\;b_{k}$, where $G\left(j,k\right)=\frac{1}{g}\min\left(j,k\right)\left(g-\max(j,k)\right)\geq 0$ is the Green’s function of $-\Delta^{2}$ under homogeneous boundary conditions. Because G≥0 and $\sum_{k=1}^{g-1}G\left(j,k\right)=\frac{1}{2}j\left(g-j\right)$ (the solution of $-\Delta^{2}u=1$, $u_{0}=u_{g}=0$), we obtain $|e_{j}|\leq M\sum_{k}G\left(j,k\right)=\frac{1}{2}M\;j\left(g-j\right)$, and maximising j(g−j) at j=g/2 yields the second inequality.    □

**Corollary** **1** (Exactness on affine signals). *If x is affine on $\left[t_{i},t_{i+1}\right]$ then M=0 and the reconstruction is exact on that interval. In particular, any policy that samples at least its first and last index reconstructs a globally affine signal with RMSE=0.*

**Proposition** **1** (Grammar-wide worst-case guarantee). *Let $M_{⋆}=\max_{s}\left|{\left(\Delta^{2}x\right)}_{s}\right|$ over the whole series and let π be any policy expressible in the grammar of Section 3.3 whose samples bracket every reconstructed index. Since the period clamp forces every gap to satisfy $g\leq p_{\max}$,*

(14) $$\mathrm{RMSE}\left(\pi \right)\;\leq {\;∥e∥}_{\infty }\;\leq \;\frac{M_{⋆}\;p_{\max}^{2}}{8}.$$

**Proof.**  Every reconstructed index lies in a gap of length $g\leq p_{\max}$, so Lemma 1 gives $|e_{t}|\leq M_{⋆}g^{2}/8\leq M_{⋆}p_{\max}^{2}/8$ pointwise. The bound on RMSE follows from $\mathrm{RMSE}\left(\pi \right)={\left(\frac{1}{N}\sum_{t}e_{t}^{2}\right)}^{1/2}\leq {∥e∥}_{\infty }$.    □

**Proposition** **2** (Monotonicity of the running policy archive). *Let $A_{r}$ denote the non-dominated archive obtained after round r by pooling all policies generated up to that round and removing dominated policies. Let $\mathrm{HV}(A_{r})$ be its dominated hypervolume with respect to a fixed reference point. Then*

$$\mathrm{HV}\left(A_{r+1}\right)\geq \mathrm{HV}\left(A_{r}\right)$$

*for every round r. Moreover, if round r+1 contributes at least one policy that is not dominated by $A_{r}$ and dominates a region of positive area, then the inequality is strict.*

**Proof.**  By construction, the candidate set at round r+1 contains all policies already available at round *r*, together with the newly generated policies. Removing dominated policies does not change the dominated region in the (d,RMSE) plane. Therefore the dominated region of $A_{r}$ is contained in the dominated region of $A_{r+1}$, so the hypervolume cannot decrease. If a newly added non-dominated policy dominates a region of positive area not already dominated by $A_{r}$, the dominated region strictly expands, and the hypervolume strictly increases.    □

This proposition does not guarantee that an LLM will find an improving policy in every round; rather, it shows that the archive-based feedback loop has a one-sided improvement property: useful policies are retained, harmful policies are discarded, and any genuinely new non-dominated policy strictly expands the front.

Two consequences follow. First, Equation (14) is a structural guarantee: it depends on the grammar (through $p_{\max}$) rather than on any particular rule set, and therefore holds uniformly for the LLM-generated policies, the random-search policies, and the fixed-rate baseline alike. It is a worst-case bound and is typically far from tight; the trailing segment beyond the last sample, if any, is a zero-order hold and instead obeys the weaker first-order bound $|e_{t}\left|\;\leq \sum |\Delta x\right|$ over that segment. Second, and more importantly, Lemma 1 shows that reconstruction error is governed by the signal’s second-order behaviour (its curvature $\Delta^{2}x$), whereas the grammar’s features (6)–(8) are all first-order (differences and slopes), exposing curvature only indirectly through ewm_var. A causal, first-order policy therefore has only limited access to the quantity that actually drives error, which bounds how far any first-order policy—LLM-generated or otherwise—can push the frontier.

## 4. Results

We report results for four contemporary LLMs—Claude Fable 5, Claude Opus 4.8, ChatGPT 5.4, and ChatGPT 5.5—each run with five independent seeds under the agentic loop of Section 3.5. Unless stated otherwise, a policy front is summarised by its hypervolume in the (d,RMSE) plane (Section 3.7; larger is better), and all dispersion is taken across the five seeds per model.

### 4.1. Policies Improve Across Optimization Rounds

**Figure 3:**
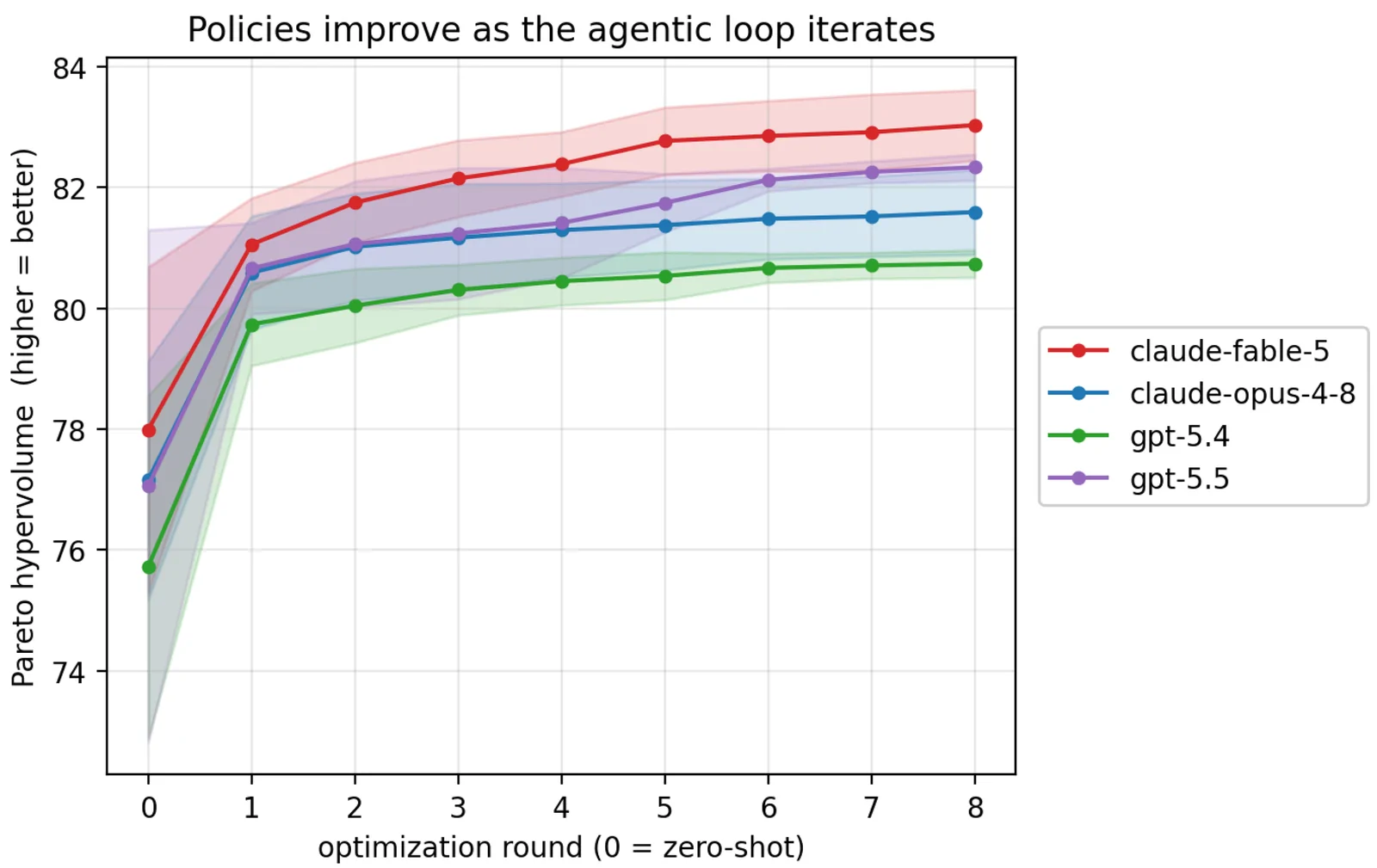
Running Pareto hypervolume of the generated policies as a function of optimization round (round 0 is zero-shot). Lines are the mean across five seeds per model; shaded bands are ±1 standard deviation. Feedback rounds add non-dominated policies beyond the zero-shot front, with most of the archive gain realised in the first feedback round.

Figure 3 traces the running Pareto hypervolume as a function of the optimization round, where round 0 is the zero-shot generation and rounds 1–8 apply the evaluate-and-improve feedback. Consistent with Proposition 2, the running policy archive expands substantially after feedback is introduced: later rounds add non-dominated policies beyond the zero-shot front, increasing the archive hypervolume for every model, and the improvement is front-loaded: the largest single gain occurs at the first feedback round and the fronts approach a plateau by rounds four to five. Averaged over seeds, the mean hypervolume rises from 77.99 to 83.03 for Fable 5, from 77.15 to 81.59 for Opus 4.8, from 75.72 to 80.74 for ChatGPT 5.4, and from 77.06 to 82.33 for ChatGPT 5.5. The between-seed dispersion also contracts sharply after the first round—for example, the standard deviation for ChatGPT 5.5 falls from 4.24 at round 0 to 0.22 at round 8—indicating that the feedback loop not only improves the fronts but also makes the outcome markedly more consistent than zero-shot generation.

Whether R=8 was a binding constraint is settled by an explicit ablation on *R*. The prompt issued at round *r* is a function only of the signal summary, the seed-selected variant, and a feedback digest built from the policies generated in rounds earlier than *r*; nothing in rounds 0,…,r depends on how many rounds will follow, since *R* enters the procedure of Section 3.5 solely through the termination condition. A run configured with R=r therefore ends in exactly the state that a run configured with R=8 passes through after its *r*-th round, and the ablation can be read off the recorded trajectories without further generation. Table 2 reports it for R=0,…,8 with K=12 held fixed; the hypervolumes recomputed for the table agree exactly with those logged at run time.

Raising *R* from 0 to 8 raises the mean hypervolume over the twenty runs from 76.98 to 81.92, a gain of 4.94 units (6.4%), at a cost of one additional API call and K=12 additional evaluations per round. The return is steeply concave in both quality and money. The first feedback round supplies 71% of the total gain for $0.08 per run; the remaining seven rounds supply the other 29% for $0.58, that is $0.023 per hypervolume unit at round one against $0.41 per unit thereafter. Five rounds supply 90% of the gain and six supply 95%; the eighth contributes 0.073 units, 0.09% of the level reached. Eleven of the twenty runs are within 1% of their own R=8 hypervolume by R=2, and all twenty by R=6. The between-run standard deviation falls from 2.92 at R=0 to 0.88 at R=1 and remains near 0.95 thereafter, so the extra rounds buy consistency almost entirely at the first one. The same shape holds at the operating points that matter for deployment: the best RMSE under a 10% duty budget falls from 42.07 to 39.37 and under a 20% budget from 27.45 to 23.58, with roughly half of each improvement realised by R=2 and over 80% by R=5.

The comparison against the control sharpens the same point. The budget-matched random search of Section 3.6 evaluates 300 policies at every truncation point—at least 2.8× the LLM evaluation count at R=8 and 25× at R=0—so the control is generous rather than matched at small *R*. Its mean hypervolume over the twenty runs is 79.86—the mean rather than the median of 80.25 used elsewhere, so that it is comparable with the means in Table 2—and the looped archive exceeds it already at R=1: two API calls, 24 evaluated policies and $0.15. We therefore read R=8 as comfortably past the knee rather than as a binding constraint. A practitioner paying per call can stop at R=5, saving 36% of the spend for 0.39% of the hypervolume. The ablation does not speak to R>8, but the final three rounds together contribute 0.32 units between them, against a between-seed spread of 80.40–83.74 (Section 4.5), which leaves little room for larger values to matter.

The proposal side bounds what a larger *K* could contribute. Of the K=12 policies generated in each feedback round, the number entering the running non-dominated front falls from 7.7 in the first feedback round to 1.4 in the last, that is from 64% to 12%. Later proposals arrive already dominated, so raising *K* would mostly add policies the archive discards. Unlike *R*, *K* cannot be ablated from the recorded runs, since lowering it discards proposals the later rounds were conditioned on and raising it requires new generation; Section 4.2 therefore reports runs at matched evaluation budget with *K* and *R* traded against one another.

### 4.2. Behaviour at a Matched Evaluation Budget

The ablation above varies *R* with *K* fixed, so it varies the budget as well as the schedule. The complementary question is how the loop behaves when the budget is held fixed and only its schedule changes. A run evaluates (R+1)K policies, so we chose the three factorisations of 108 that keep the published K=12 arm in the set: (R,K)=(8,12), (5,18) and (3,27), spending the same 108 evaluations in nine, six and four API calls respectively. Two models (Claude Fable 5 and ChatGPT 5.5) were run with five seeds per arm, paired by seed as elsewhere.

All three arms were generated in August 2026, including a fresh (8,12) arm. This matters: re-running (8,12) on the same day as the new arms returned a mean hypervolume 0.44 lower than the July runs for Fable 5 and 0.33 lower for ChatGPT 5.5 (paired Wilcoxon p=0.19 in both cases). Neither shift is significant at n=5, but both point the same way, and comparing August arms against a July reference would have confounded the schedule with the date. The arms below therefore use the contemporaneous (8,12) runs; the July runs remain the basis of every other table in this paper and are unchanged.

Table 3 reports the outcome. The two arms that spend the budget in fewer, larger rounds attain a slightly higher mean hypervolume than the published schedule—82.87 at both (3,27) and (5,18) against 82.30 at (8,12)—and are indistinguishable from one another (−0.003 units, p=0.77). The Friedman omnibus across the three arms does not reject interchangeability at the conventional level ($\chi^{2}=5.60$, p=0.061). Paired Wilcoxon tests give p=0.010 for (5,18) against (8,12) and p=0.037 for (3,27) against (8,12), but under Holm correction for the three comparisons only the first survives, and neither model reaches significance on its own five seeds. We therefore do not claim that fewer, larger rounds are better. What the experiment establishes is the weaker and more useful statement: spending the same budget in four calls rather than nine costs nothing measurable, so the loop is robust to how the budget is scheduled.

Two observations qualify this. First, the ordering reverses at the tightest operating point: the nine-call schedule attains the best RMSE within a 10% duty budget (35.43 against 36.76 at (3,27)), while the fewer-call arms lead at d≤0.20. More feedback rounds appear to help precisely where the frontier is hardest to move, which is consistent with the curvature-limited analysis of Section 3.8. Second, the saving is in calls rather than in money: because the total number of generated policies is fixed at 108 and output tokens dominate the bill, the estimated cost per run varies by under 5% across the three arms. The four-call schedule is preferable where per-request latency or rate limits bind, not where the token bill does.

ChatGPT 5.5 occasionally returned more policies than requested, so the realised budget is 108 exactly for every Fable 5 run and 108–111 for ChatGPT 5.5; the arms are matched nominally rather than to the policy. Two ChatGPT 5.5 runs were repeated after a round returned malformed JSON that the parser rejected; the repeats are infrastructure failures rather than experimental outcomes, and only the completed runs are reported.

### 4.3. The Loop Improves on Zero-Shot and on Random Search

**Figure 4:**
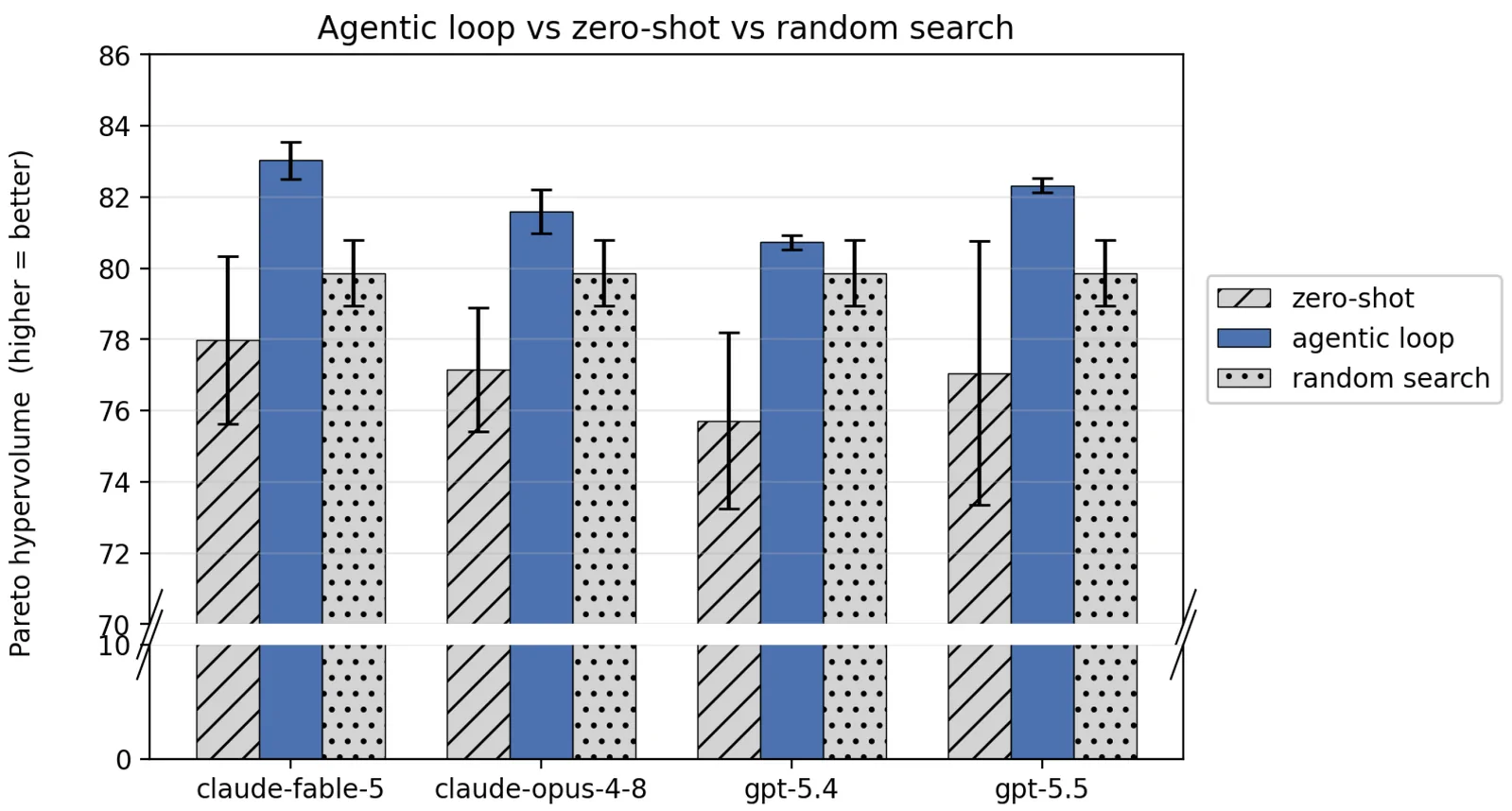
Pareto hypervolume of the looped front against the zero-shot front and budget-matched random search, per model. Bars are the mean across five seeds; error bars are 95% confidence intervals. The *y* axis is broken between 10 and 70: every value lies between 74 and 84 on a scale whose reference point is 100, so a full-range axis would compress all twelve bars into the top fifth of the panel. The lower panel retains the zero baseline. The looped front is highest for every model.

Table 4 and Figure 4 compare, for each model, the final looped front against two references: its own round-0 zero-shot front, and the budget-matched random search over the identical grammar. The looped front has a higher median hypervolume than the zero-shot front for every model, and exceeds the random-search median for every model as well.

Because each model contributes only five seeds, a paired Wilcoxon signed-rank test at the model level cannot reach p<0.05 (its attainable minimum at n=5 is 0.0625); the per-model *p*-values in Table 4 sit at or just above that floor and are reported for completeness. We therefore test the central claim—that the loop improves on its references—by pooling all twenty runs. Pooled across models and seeds, the looped front (median hypervolume 82.11) significantly exceeds both the zero-shot front (median 77.29; Wilcoxon $p<10^{-5}$) and the budget-matched random search (median 80.25; Wilcoxon $p<10^{-5}$). The improvement over unguided search of the same policy space—under the policy-evaluation control described above—supports the role of the guided, feedback-driven procedure rather than exploration alone.

### 4.4. Energy–Fidelity Frontier and Budget-Wise Fidelity

**Figure 5:**
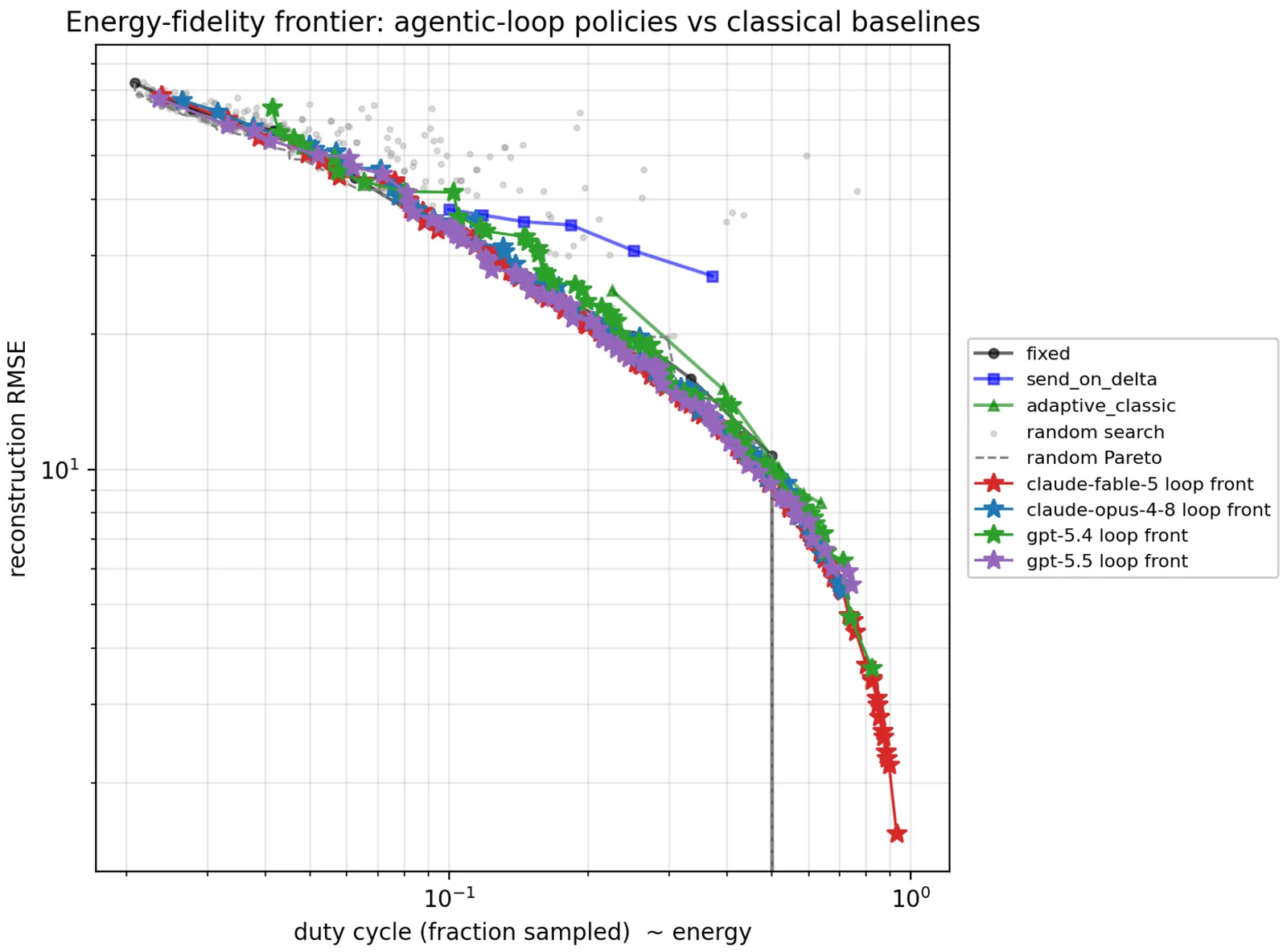
Energy–fidelity plane on logarithmic axes: pooled looped fronts of the four models (coloured stars) against the fixed-rate, delta-triggered adaptive-period, and prediction-based baselines and the random-search cloud on the Beijing PM_2.5_ series. Lower and to the left is better. The looped fronts track and, at d≳0.2, sit on the tight lower envelope of the classical frontier.

Figure 5 plots the pooled looped fronts of all four models against the classical fixed-rate, delta-triggered adaptive-period, and prediction-based baselines and the random-search cloud, on the full (d,RMSE) plane. The looped fronts track the tight lower-left envelope of the classical frontier across the operating range and extend it into the very-low-error, high-duty region.

Table 5 quantifies this by reporting, at fixed duty-cycle budgets, the best RMSE each model’s looped front attains, against the budget-matched random search and the three classical families, each with a 95% confidence interval across seeds where one applies. At the two largest budgets (d≤0.30 and d≤0.50) all four models improve on every alternative—at d≤0.30 the looped RMSE ranges from 15.98 (Fable 5) to 17.69 (ChatGPT 5.4) against 19.82 for the best classical baseline and 20.57 for random search, and at d≤0.50 from 9.75 to 10.39 against 10.73 and 11.74. At the intermediate budgets the stronger models (Fable 5 and ChatGPT 5.5) continue to lead while Opus 4.8 converges to the classical frontier and ChatGPT 5.4 falls behind it.

Two features of the table qualify the picture and are visible only once dispersion and the classical columns are shown. First, at the tightest budget (d≤0.10) no model separates from random search, and the intervals show why the margin should not be read as one: Fable 5 attains 35.29 [34.49,36.08] against 35.31 [34.51,36.11] for random search, so the two are indistinguishable rather than narrowly separated. Second, fixed-rate sampling is a stronger reference than the random-search control at d≥0.20, attaining 24.99, 19.82 and 10.73 against the control’s 25.09, 20.57 and 11.74; the loop’s margin at those budgets is therefore measured against fixed-rate rather than against random search, and ChatGPT 5.4 does not attain it at d≤0.20 or below. The advantage of the loop is thus real but budget- and model-dependent—clearest where a few extra samples can be spent adaptively, and absent at the most aggressive subsampling, consistent with the curvature-limited bound of Section 3.8 and the high short-lag autocorrelation of the signal (Section 3.4).

### 4.5. Comparison Across Models

The four models occupy overlapping regions of the tradeoff, with median looped hypervolumes of 82.84 (Fable 5), 82.39 (ChatGPT 5.5), 81.44 (Opus 4.8), and 80.76 (ChatGPT 5.4). A Friedman omnibus test across the four models, paired by seed, rejects the hypothesis that they are interchangeable ($\chi^{2}=12.12$, p=0.0070).

That is all these five seeds support. The omnibus test establishes only that some difference exists among the four; it does not license any statement about which model is better than which. At n=5 the attainable minimum of the paired Wilcoxon is 0.0625, so no pairwise comparison could reach conventional significance even if the underlying difference were large, and we therefore report no pairwise *p*-values and assert no ordering. The per-seed spread illustrates why such caution is warranted: the four models span 80.40–83.74 across all twenty runs, and the ranges of Fable 5 (82.41–83.74) and ChatGPT 5.5 (82.07–82.59) sit close enough that seed-level variation, not model identity, could plausibly account for their apparent separation.

We therefore read Table 4 and Table 5 as evidence that every model benefits from the loop and that all four cover a broadly similar region of the tradeoff, not as a leaderboard. Establishing a defensible ordering would require substantially more seeds per model, which we identify as future work in Section 5.1.

### 4.6. Generalisation Across Sensor Channels

All policies so far were compiled on the PM_2.5_ series. To test whether they carry beyond it, we replayed every LLM-generated policy—unchanged, with no model re-invoked and no threshold re-tuned—on the four further channels recorded synchronously in the same dataset over the same 43,824 h: temperature, dew point, barometric pressure, and cumulated wind speed. Random search and the classical baselines were recomputed natively on each channel. Because RMSE scales differ between channels, the hypervolume reference is set per channel to $\left(d_{\mathrm{ref}},{\mathrm{RMSE}}_{\mathrm{ref}}\right)=\left(1.0,\sigma_{x}\right)$; values are therefore comparable across methods within a channel, but not between channels.

**Figure 6:**
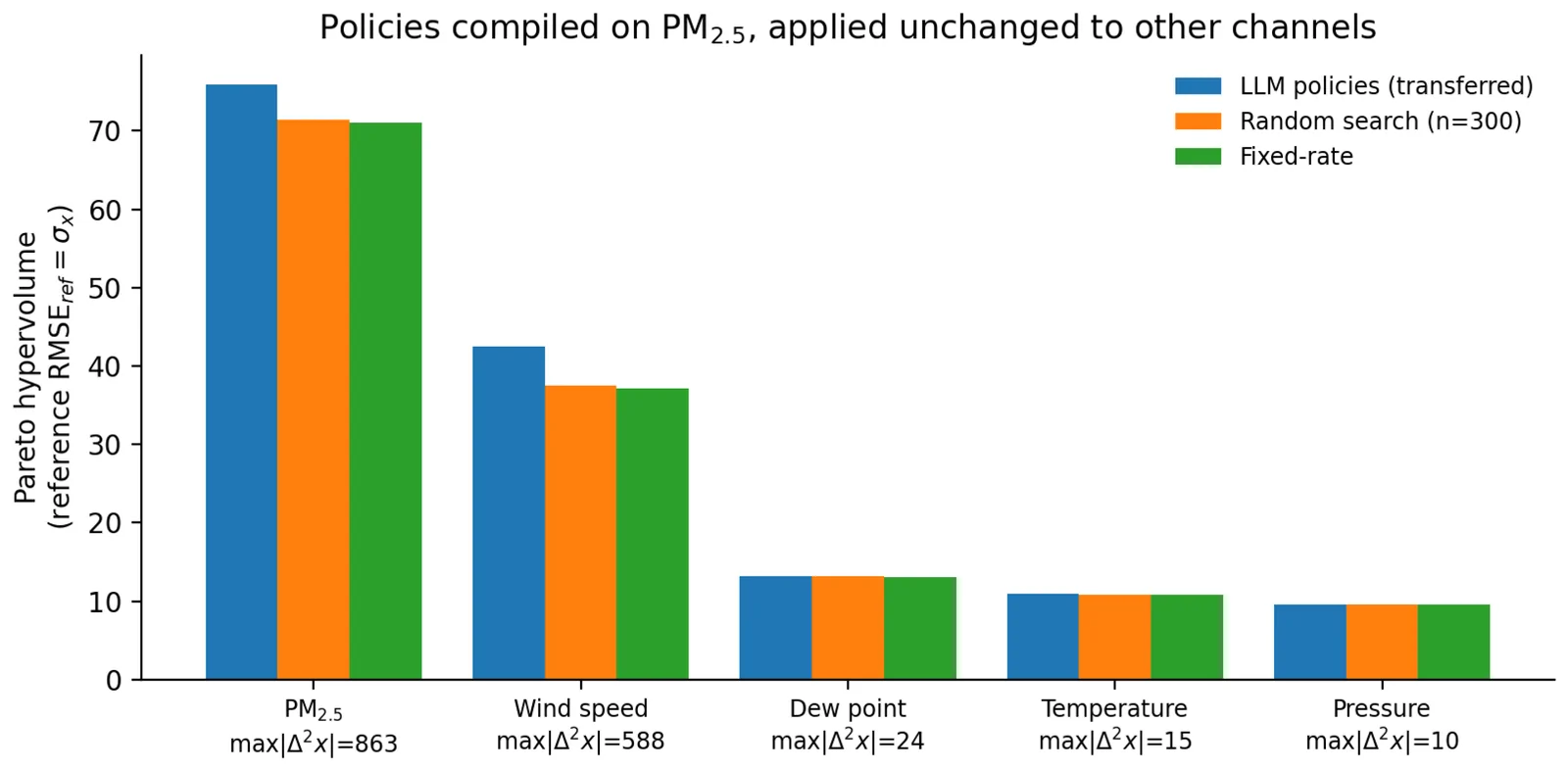
Policies compiled on PM_2.5_ applied unchanged to four further sensor channels, against random search and fixed-rate sampling computed natively on each. Channels are ordered by maximum discrete curvature. The advantage of adaptive sampling tracks curvature, as Lemma 1 predicts.

Table 6 and Figure 6 report the outcome, ordered by maximum discrete curvature $M_{⋆}=\max_{s}\left|{\left(\Delta^{2}x\right)}_{s}\right|$. The transferred policies retain a clear margin over random search on the two high-curvature channels (+4.53 on PM_2.5_, +4.91 on wind speed) but not on the three smooth ones, where the margin falls to between +0.02 and +0.08.

This is what Lemma 1 predicts, and the final two columns make the point quantitative. The largest hypervolume any policy could attain is ${\mathrm{RMSE}}_{\mathrm{ref}}$; subtracting what fixed-rate sampling already achieves leaves the room available to any adaptive policy. That room is 19.93 and 12.93 units on PM_2.5_ and wind speed, but only 0.74–1.39 units on pressure, temperature and dew point—a contraction of 15–27×. Temperature has curvature $M_{⋆}=15$ against 863 for PM_2.5_: since $|e_{t}|\;\leq M_{⋆}g^{2}/8$, piecewise-linear reconstruction is already close to exact at any admissible period, so fixed-rate sampling captures 89% of the attainable hypervolume unaided and no policy—ours, random, or classical—can separate from the others. Where room does exist, the compiled policies claim a substantial share of it: 25% on PM_2.5_ and 41% on wind speed, against 6–10% on the smooth channels.

The value of a compiled adaptive policy is therefore not a property of the method alone but of the signal it is deployed on, and the analysis of Section 3.8 identifies in advance the quantity that decides it. We emphasise that this experiment transfers the compiled artefacts; it does not re-run the agentic loop natively on these channels (Section 5.1).

### 4.7. Comparison Against Stronger Optimisers

Random search establishes that the loop does more than sample the grammar, but it is a deliberately unguided control. To test whether the advantage survives against optimisers that are themselves competent, we implemented two standard black-box methods over the identical grammar, scored by the identical evaluator: NSGA-II [18], with fast non-dominated sorting, crowding-distance selection, binary tournament, uniform crossover and polynomial mutation; and ParEGO [19], a multi-objective Bayesian optimiser using augmented Tchebycheff scalarisation with a fresh random weight vector each iteration, a Gaussian-process surrogate with an anisotropic Matérn kernel, and expected-improvement acquisition. NSGA-II is run for five seeds at every budget; ParEGO for five seeds at the matched budget of 108 evaluations only, since Gaussian-process fitting scales cubically in the number of observations.

NSGA-II’s configuration is stated in full, since the budget at which an evolutionary algorithm is run determines what the comparison means. It uses a (μ+λ) scheme with μ=λ=24 over a 14-gene encoding of the grammar (2 global genes plus four per rule, at most three rules), binary tournament selection on rank and crowding distance, uniform per-gene crossover, and per-gene mutation at rate 1/14: polynomial mutation with η=20 on the threshold genes, a bounded integer step in [−8,+8] on the periods, and resampling on the categoricals. The archive is unbounded—every evaluated point is retained and the hypervolume is taken over the whole set—so the archive size equals the evaluation budget by construction, and its non-dominated subset is reported below. A run at budget *B* therefore executes one initial population and ⌈(B−24)/24⌉ offspring generations: four at B=108 and 124 at B=3000. The random-search control uses the same archive convention: every sampled policy is retained and the hypervolume taken over the whole set, so its archive size likewise equals its evaluation budget.

**Figure 7:**
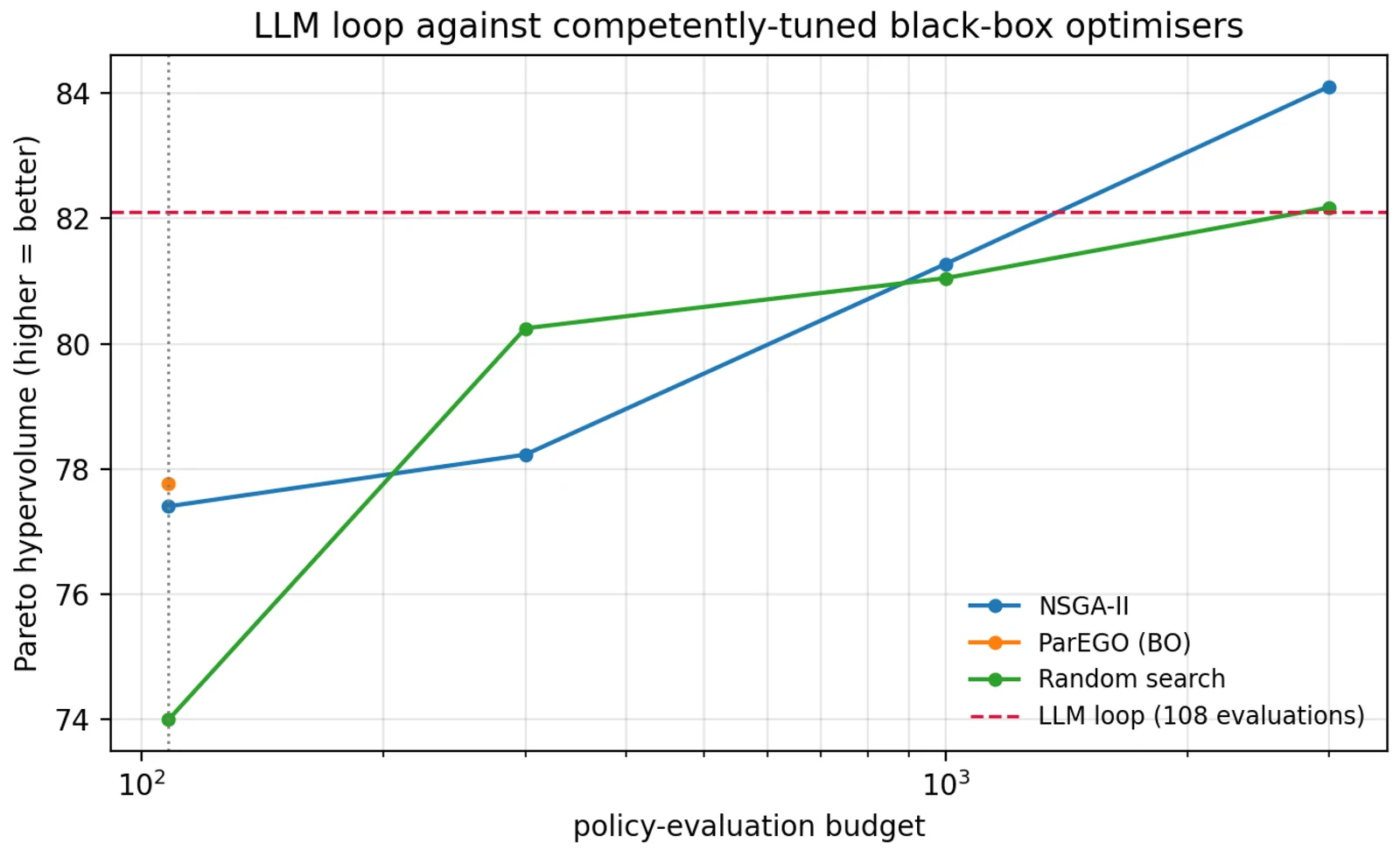
Pareto hypervolume against policy-evaluation budget for NSGA-II, ParEGO and random search over the identical grammar; the dashed line is the agentic loop at its own budget of 108 evaluations (dotted vertical line). At matched budget the loop leads every alternative; both classical optimisers overtake it only after roughly an order of magnitude more evaluations.

Table 7 and Figure 7 report the outcome. At the budget the loop actually spends—108 policy evaluations—the loop attains a median hypervolume of 82.11 against 77.77 for Bayesian optimisation, 77.40 for NSGA-II and 73.99 for random search. The advantage over unguided search is therefore not an artefact of a weak control: it persists, and indeed widens relative to the classical optimisers, when the control is a competent one.

The comparison also makes the nature of the advantage precise. Both optimisers improve steadily as evaluations are added, and both eventually overtake the loop. Read off the recorded NSGA-II trajectory rather than interpolated between budgets, the crossing occurs at approximately 1660 evaluations, at generation 69 of the B=3000 run; random search, for which no per-generation trajectory exists, reaches the same level at approximately 2810 evaluations by interpolation between budgets. Against the loop’s 108, the loop’s benefit is thus one of sample efficiency—it reaches in one budget what unguided search requires roughly 26× and a tuned evolutionary algorithm roughly 15× more evaluations to match—rather than a claim to the best front attainable given unlimited evaluations. Where a policy can be scored millions of times offline, a conventional optimiser run long enough will do better, and we report that NSGA-II attains 84.11 at 3000 evaluations. The regime in which the loop is preferable is the one where each evaluation is costly, or where no search harness has been built at all: the loop requires only a task description and the grammar, with no optimiser to be configured, encoded or tuned.

The economic comparison runs opposite to the statistical one, and Table 8 states both sides. NSGA-II issues no API calls and so costs nothing in fees; what it spends is evaluations, and through them compute: 49 s of one CPU core to reach 84.11 at B=3000, and roughly 1660 evaluations before it matches the loop at all. The loop reaches 82.11 in 108 evaluations—0.4 s of the same evaluator—for $0.73 at list prices, or $0.40 under the lower token reconstruction (Section 4.8). Which is the cheaper instrument is therefore a property of the deployment rather than of the algorithms. Where a policy can be replayed against a recorded trace millions of times, as here, the evolutionary algorithm is effectively free and eventually better, and the honest recommendation is to use it. Where each evaluation carries real cost—a hardware-in-the-loop trial, a field deployment, a long physical simulation—a dollar of API spend buys what an order of magnitude more evaluations would otherwise cost, and the ranking inverts.

One further control deserves comment, since the grammar includes a feature that is constant in the present formulation. Because time_since is identically zero at every decision point, a rule time_since <θ with θ>0 fires unconditionally and collapses the policy to a constant period, while time_since >θ never fires and is inert. Including the feature therefore injects fixed-rate policies into the random pool at no cost. It might be supposed that this weakens the control; measured, the opposite holds. Removing time_since from the random search lowers its median hypervolume from 73.99 to 70.93 at 108 evaluations and from 80.25 to 76.11 at 300, because fixed-rate sampling is itself strong on this signal. The control as published is thus the stronger of the two variants, and the reported margin over it is conservative.

Whether the metaheuristic converged has two answers, and both bear on how the comparison should be read. At the loop’s own budget it has not: 108 evaluations is four generations of a population of 24, which leaves the search far from stationary, and the five seeds span 66.75 to 77.80 against a spread of about three units for the loop. That is not a defect in the control but the substance of the claim—at a budget this small a competently configured evolutionary algorithm has not yet had time to work, and the loop has. At B=3000 it has converged. The convergence statistics below are means over the five seeds, so the final level is 83.99 rather than the median 84.11 of Table 8. The mean hypervolume reaches 95% of that level by generation 36 (888 evaluations) and 99% by generation 88 (2136); the final twelve generations together add 0.254 units, 0.30% of the level reached, with no seed gaining more than 0.755 and the final generation adding nothing measurable. The crossover reported above therefore compares the loop against a converged evolutionary algorithm, not against one still climbing.

### 4.8. Cost of Compilation and of the Deployed Artefact

Two costs decide whether the approach is practical: what the compiled policy costs on the device, and what compiling it costs offline. Both are measured here from the artefacts of the study itself.

Deployed artefact.

We encode a policy for a microcontroller target as a two-byte header (rule count and default period) followed by one record per rule: an unsigned byte each for the feature index, the comparison operator and the period, and a single-precision threshold, giving seven bytes packed or eight under four-byte alignment. The run-time state is whatever the policy’s features require: the last two samples and their indices for abs_delta, rel_delta and slope, and the running mean and variance for ewm_var, plus the scheduled period. time_since is identically zero and is not stored.

Under this encoding, every one of the 2161 policies compiled across the twenty runs occupies at most 92 bytes of flash and 28 bytes of RAM (Table 9). A decision costs at most 28 floating-point operations—at most 84 cycles on a core with a single-precision FPU, or 1120 cycles under software floating point on a core without one—and is taken once per sampling interval, which on this signal is at most once per hour. Against a deliberately modest 32 kB flash, 8 kB RAM part, the worst-case artefact occupies 0.28% of flash and 0.34% of RAM. The claim that the deployed policy is negligible on microcontroller-class hardware is therefore a measured one. Because the figures above are exact for the stated encoding rather than for any particular toolchain, we confirmed them against one. Each policy was emitted as static C data—a two-byte header followed by one record per rule, as a flexible array member so that the layout is the encoding rather than a pointer to it—compiled with GCC 13.2 at -Os for a Cortex-M4F (hard float) and a Cortex-M0+ (software float), and its section sizes read from arm-none-eabi-size at object level, which excludes startup code and the C library. The measured .rodata reproduces the encoded size exactly for every policy tested: 44 and 92 bytes for the median and largest policies, and 20, 36, 36, 36 and 44 bytes for the five best-of-study artefacts of Appendix A.

Two quantities the encoding does not capture appear once the code is compiled, and Table 9 now reports both. The rule interpreter—the code that refreshes the features, walks the rules and clamps the period—occupies 296 bytes of .text on the Cortex-M4F and 320 on the Cortex-M0+. This is not counted above because it is independent of which policy is stored and is charged once however many the device holds, but it is real flash: the complete worst-case artefact is 388 bytes on the Cortex-M4F, 1.18% of a 32 kB part rather than the 0.28% the table alone implies. In the other direction, the measured run-time state is 16 bytes of .bss against the 28 the encoding assigns, because the encoding counts $x_{\mathrm{prev}}$ and $t_{\mathrm{prev}}$ as stored slots whereas an implementation reads the previous sample from $x_{\mathrm{last}}$ before overwriting it, so neither needs to persist between decisions. The encoding is thus exact on flash and conservative on RAM, and the conclusion it supports—that the deployed artefact is negligible on microcontroller-class hardware—is unchanged. Energy and execution time on live hardware remain untested (Section 5.1).

Offline compilation.

A run issues nine API calls: one zero-shot generation and eight optimization rounds. The prompt at each round is a deterministic function of the signal summary, the seed-selected prompt variant and the feedback digest, all of which are recorded, so input tokens are counted exactly by regenerating the prompts. Completions are reconstructed from the logged policies, which recovers the JSON payload but not any whitespace, code fencing or prose that the parser discarded; output tokens are therefore reported as a range whose lower end is the compact payload and whose upper end is the same payload indented. Counts use a single byte-pair tokeniser for all four models, so the figures for the Claude models are approximate to within a few percent. Token counts are converted to currency at provider list prices as of 11 August 2026 (standard non-batch tier; prompt caching was not enabled): $10.00/$50.00 per million input/output tokens for Claude Fable 5, $5.00/$25.00 for Claude Opus 4.8, $5.00/$30.00 for ChatGPT 5.5 and $2.50/$15.00 for ChatGPT 5.4. Because the output-token count is a reconstruction, the cost inherits the same lower and upper bounds; batch submission or prompt caching would reduce both. This is the same basis used for the cost column of Table 2.

Table 10 reports the outcome. A complete compilation—nine calls, eight feedback rounds, 108 evaluated policies—consumes between roughly 18,000 and 39,000 tokens depending on the model, which at list prices is between $0.24 and $1.02: under one dollar even for the most expensive model at the pessimistic end of the reconstruction. The whole study, twenty runs across four models, cost 180 calls, 182,445 input tokens, between 244,094 and 478,244 output tokens, and between $7.96 and $14.62 in total. For a practitioner the operative figure is the per-run one: compiling a policy for a new signal is a sub-dollar operation, incurred once, against a deployed artefact that then costs nothing.

The two costs are asymmetric in the way the approach intends. Compilation is a one-off charge of a few tens of thousands of tokens, incurred once, offline, before deployment; the artefact it produces then runs for the lifetime of the device at a cost of tens of bytes and tens of operations per sample. Wall-clock latency of the loop was not instrumented, and is in any case immaterial to the deployed system, since no model is invoked after compilation.

### 4.9. Sensitivity to the Grammar’s Fixed Parameters

Two constants in Section 3.3 were fixed a priori: the smoothing factor α=0.2 of the exponentially weighted variance, and the period cap $p_{\max}=48$. Neither was tuned, and neither was justified in the original submission. We therefore re-scored all 2161 policies compiled across the twenty runs under every combination of α∈{0.05,0.10,0.20,0.40,0.80} and $p_{\max}\in \left\{12,24,48,96,168\right\}$, using the same deterministic evaluator and without issuing any further API calls. At the published setting the replay reproduces the recorded scores exactly, with a maximum absolute deviation of $7.1\times 10^{-15}$ across all policies, confirming that the sensitivity grid and the published results are produced by the same evaluator. Table 11 reports the outcome.

Smoothing factor.

Over α∈[0.05,0.40] the pooled front hypervolume varies by 0.59 units, from 81.62 to 82.21, a spread of 0.7%, and the best RMSE within a 0.20 duty budget varies by less than 0.5, from 22.65 to 23.09. The results are therefore insensitive to α across more than an order of magnitude. Only at α=0.80, where the variance estimate tracks individual samples too closely to carry information about local volatility, does performance degrade materially: hypervolume falls to 79.80 and the best RMSE at d≤0.20 rises to 26.86, a 17% increase. The published value is not the argmax—α=0.10 is nominally better by 0.10 hypervolume units, which is within the run-to-run variation of Section 4.5—so the reported results do not rest on a favourable choice of this constant.

Period cap.

Reducing the cap costs fidelity: at the published α, hypervolume falls from 82.11 to 81.57 at $p_{\max}=24$ and to 79.65 at $p_{\max}=12$, since a tighter clamp forecloses the sparse end of the frontier. Raising the cap changes nothing at all: the $p_{\max}=96$ and $p_{\max}=168$ columns are identical to $p_{\max}=48$ in every entry, for every α.

That identity is weaker evidence than it looks. The longest period requested by any of the 2161 compiled policies is exactly 48—at the cap—so re-scoring under a larger cap cannot change any score: no policy in the archive asks for a period the larger cap would newly permit. The columns therefore show that the compiled archive does not use periods beyond two days, not that a loop run under a larger cap would fail to find such policies. A maximum coinciding with the cap is consistent with a binding constraint; settling it requires re-running the generation loop, which we identify as future work in Section 5.2.

Two considerations nonetheless support $p_{\max}=48$ for the operating range this study reports. First, a period of 48 already admits duty cycles down to 1/48≈0.021, about five times below the tightest budget in Table 5; a larger cap would extend the frontier into a region the evaluation does not report. Second, the worst-case guarantee of Proposition 1 scales as $p_{\max}^{2}$, so moving to $p_{\max}=168$ would weaken the structural bound by a factor of roughly 12 in exchange for frontier extension that the present archive does not exploit.

### 4.10. Chronological Train/Test Split

The results above are in-sample: a policy’s thresholds were chosen against scores measured on the series it is then reported on. Section 3.4 argues that the grammar is too small to memorise a 43,824-point trace, but an argument is not a measurement, and a policy need not memorise a trace to encode statistical quantities peculiar to it. We therefore re-ran the loop under a chronological split.

The cut is a calendar-year boundary: the loop compiles on 2010–2013 (35,064 h) and every reported score is taken on the held-out 2014 segment (8760 h). A whole year on each side matters here, because PM_2.5_ in Beijing is dominated by a winter heating cycle; an 80/20 cut by index would have divided that cycle and confounded the split with seasonality. The two segments are closely matched in level and spread—mean 98.3, standard deviation 90.2 on the training segment against 98.0 and 93.8 on the held-out one.

Three restrictions make this a genuine split rather than a re-scoring. The eight prompt scalars of Section 3.5 are computed from the training segment alone, which closes the principal channel by which the held-out year could reach a compiled policy in a method that is statistics-informed by construction. The evaluator inside the loop sees the training segment only, so every score the optimizer is shown—the running front, the classical targets, the underperforming examples—is measured there. And the random-search control and the three classical families are recompiled on the same training segment, so the comparison stays like for like. One further symmetry is needed for the comparison to be fair: the loop’s front is selected on the training segment, so the control’s front is selected there too. Scoring all 300 random draws on the held-out year would let the control choose with knowledge the loop is denied.

**Figure 8:**
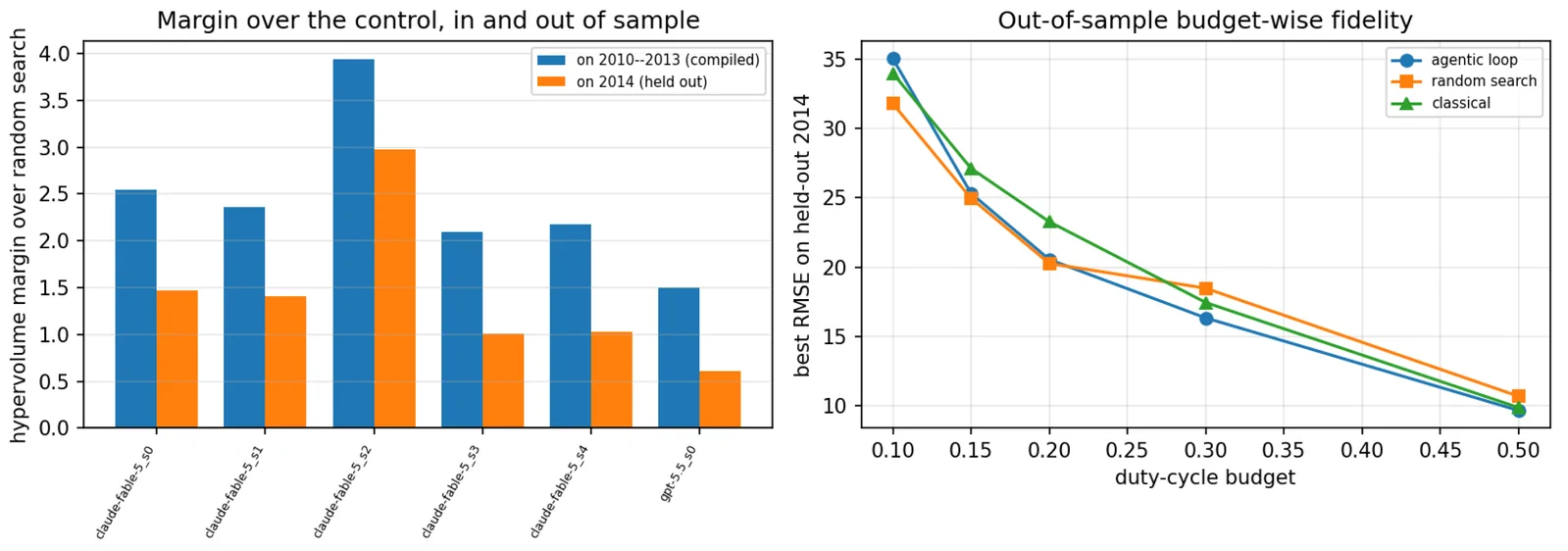
Chronological split. Left: the loop’s hypervolume margin over the budget-matched random search, per run, on the segment it was compiled on and on the held-out 2014 segment. The margin is positive in every run on both, and smaller on the held-out one. Right: best RMSE within each duty-cycle budget on the held-out segment, all fronts selected on the training segment.

Table 12 and Figure 8 report the outcome over six runs. Absolute levels are higher on the held-out segment for every method—the loop rises from 82.02 to 82.85, random search from 79.58 to 81.44, the classical families from 80.70 to 81.97. That is a property of the segment measured against a fixed reference point, not a statement about any method, and in particular it is not evidence that the in-sample numbers were pessimistic. Levels are therefore not comparable across the two segments, and we read the split through the margin over the controls, which is.

That margin survives and contracts. Against the budget-matched random search the loop leads by 2.44 hypervolume units on the segment it was compiled on and by 1.42 on the held-out one, positive in all six runs (paired Wilcoxon p=0.031, the attainable minimum at n=6); against the classical families the figures are 1.32 and 0.88, again six of six (p=0.031). Roughly 42% of the advantage over unguided search does not transfer to the held-out year, and the contraction itself is consistent across runs (−1.02 units, six of six, p=0.031). Some part of the in-sample margin was therefore trace-specific; the majority of it was not. Selecting the front on the training segment rather than the held-out one costs a further 0.10 units, which we absorb rather than report around, since it is what a deployment incurs.

Table 13 shows where the surviving margin lives, and the answer sharpens the picture rather than softening it. Out of sample the loop leads only at the two loosest budgets—16.33 against 18.47 for random search at d≤0.30 and 9.64 against 10.66 at d≤0.50—while at d≤0.10 it trails the control substantially (35.06 against 31.81) and at d≤0.15 and d≤0.20 narrowly. This is the same budget dependence reported in Section 4.4 and explained by the curvature bound of Section 3.8, but out of sample it is more pronounced: what the loop compiles for the tight end of the frontier is the part that transfers least well. A practitioner operating below a 0.20 duty cycle on a signal of this kind should not expect the compiled policy to beat unguided search on unseen data.

Two disclosures. The split arm comprises five Claude Fable 5 seeds and one ChatGPT 5.5 seed; the remaining ChatGPT 5.5 seeds were not completed within the available API budget, so the experiment is effectively single-model with one cross-model check, and we do not read it as a comparison between models. Two of the six runs evaluated fewer than the nominal 108 policies—84 and 96—because individual rounds returned JSON the parser rejected, giving 612 compiled policies in total; the shortfall is an infrastructure failure rather than an experimental outcome, and it works against the loop by reducing its budget relative to the 300-policy control.

### 4.11. Summary of Findings

Across all twenty runs the loop improves on both its zero-shot output and unguided random search (pooled Wilcoxon $p<10^{-5}$ in both cases), leads every alternative optimiser at matched budget, and transfers to further channels in the way the curvature bound predicts. Under a chronological split it retains a margin over the control in every run, though roughly 42% of that margin does not transfer to the held-out year. The gain is budget-dependent and vanishes at the tightest subsampling; the resulting policies remain compact and free of any on-device model.

## 5. Discussion

Three findings from Section 4 bear on how the approach should be used rather than on whether it works. First, the loop’s advantage is one of sample efficiency and not of the best attainable front: given enough evaluations a conventional optimiser overtakes it (Section 4.7), so the regime in which it pays is the one where each evaluation is costly or no search harness exists. Second, the advantage is a property of the signal as much as of the method—it tracks curvature, and on smooth channels no policy of any kind separates from fixed-rate sampling (Section 4.6). Third, what is deployed stays negligible throughout: a policy of at most 92 bytes, a 296-byte interpreter charged once whatever the policy, 16 bytes of RAM and tens of operations per decision, together 1.18% of a 32 kB part in the worst case (Section 4.8), so the gain is not bought with on-device cost. Together these delimit where compiling a policy is worth the effort: signals with appreciable curvature, deployments where per-evaluation cost is real, and applications where an interpretable artefact matters.

### 5.1. Limitations

Several limitations qualify the scope of our claims.

Single generating signal: Policies are compiled on the Beijing PM_2.5_ series alone. Section 4.6 extends the evaluation to four further channels and shows that the margin over unguided search tracks signal curvature as the analysis predicts, but the agentic loop itself was not re-run on those channels: we establish that the compiled artefacts generalise in a predictable way, not that the procedure confers the same advantage when applied natively to a different signal. All five channels also share one meteorological context, one measurement site and one sampling rate. Whether the loop reproduces its gain when run natively on vibration, industrial telemetry, or biomedical signals at other rates remains to be tested.
Scope of the chronological split: Section 4.10 holds out one year of one series, at one cut. It establishes that the margin over unguided search survives a genuine temporal split on this signal, and measures how much of it does not, but a single held-out year cannot distinguish a margin that degrades with distance from the training segment from one that degrades with any change of segment. Nor was the split re-run at other cut points, on the four further channels, or on more than one model at five seeds.
Limited seeds per model: Each model is run with five independent seeds. This is enough to establish the loop’s advantage over its references by pooling across all twenty runs, but five seeds is too few for a paired test at the model level: the attainable minimum of the paired Wilcoxon at n=5 is 0.0625, so no per-model comparison can reach conventional significance. We therefore do not claim a reliable pairwise model ranking; the cross-model view of Section 4.5 is read as coverage of the tradeoff space rather than as a leaderboard. Establishing a definitive order would require more seeds per model with reported dispersion. The loop’s other two structural constants are now characterised unevenly. *R* is ablated exactly over 0,…,8 in Section 4.1, which shows the marginal return close to zero well before the end of a run; that ablation cannot, however, speak to R>8, since it truncates recorded trajectories rather than extending them. For *K*, Section 4.2 reports a matched-budget trade against *R* on two of the four models, which finds no penalty from spending the budget in fewer, larger rounds; it does not sweep *K* at fixed *R*, so what a larger total budget would add remains bounded by the per-round front growth rather than measured.
Scale of the search-budget sweep: The headline random-search control uses at least as many policy evaluations as the corresponding LLM run, with a minimum of 300 policies. Section 4.7 additionally sweeps random search and NSGA-II to 3000 evaluations, which is where both overtake the loop, so the sample-efficiency claim rests on measured crossover points rather than on an untested extrapolation. We did not extend the sweep to $10^{4}$ evaluations or beyond, and ParEGO was run only at the matched budget of 108 because Gaussian-process fitting scales cubically in the number of observations; how a Bayesian optimiser behaves at large budgets on this problem is therefore not characterised here.
Use of summary statistics: The prompt supplies signal-level summary statistics, including mean, standard deviation, autocorrelations, median change, and 95th-percentile change. This is not training on the raw series, and no weights are updated, but it is nonetheless dataset-specific information. The approach is best described as training-free but statistics-informed rather than fully signal-agnostic.
Reconstruction operator and grammar: We fix a single, model-free reconstruction operator, namely piecewise-linear interpolation, and a first-order rule grammar. As Section 3.8 shows, reconstruction error is governed by the signal’s second-order curvature, to which the grammar’s first-order features have only indirect access through ewm_var. Section 4.6 measures this ceiling per channel, and finds that on temperature fixed-rate sampling already captures 89% of the attainable hypervolume, leaving 1.39 units to any adaptive policy against 19.93 on PM_2.5_. The headroom available to a first-order policy is therefore a property of the signal, and it can be estimated in advance from the curvature. Richer reconstructions, such as spline or total-variation methods, or curvature-aware features could raise that ceiling, at the cost of additional on-device state.
Trace-driven evaluation: Policies are replayed offline on the recorded signal. Duty cycle is a hardware-independent energy surrogate that counts sampling events and abstracts away per-sample and radio energy, wake-up latency, and quantisation. The deployed artefact’s memory footprint is measured with a cross-compiler in Section 4.8, but no policy has been executed on physical silicon: validation on live battery-powered hardware, measured execution time, and a more detailed energy model are left to future work.
Model reproducibility: The specific model versions are proprietary and may change over time. We report the model names, access period, and generation settings in Section 3.5, but exact regeneration of the same outputs cannot be guaranteed for hosted models. Section 4.2 quantifies this for the (8,12) configuration: re-running it in August 2026 returned a mean hypervolume 0.44 (Fable 5) and 0.33 (ChatGPT 5.5) below the July runs, neither shift significant at n=5 but both in the same direction. Comparisons across dates should therefore be avoided, and the experiments of Section 4.2 were run with a contemporaneous reference for that reason.
Scope of the optimiser comparison: The random-search control samples uniformly from the full grammar, including time_since, which is constant in the present one-step formulation; Section 4.7 shows that removing it makes the control weaker rather than stronger, so this choice is conservative. It remains a baseline for unguided grammar exploration only, and Section 4.7 therefore adds NSGA-II and ParEGO over the identical grammar. Two families remain untested: grammar-aware search operators that exploit the rule structure directly, and surrogate models other than a Gaussian process, such as tree-based or trust-region methods, which scale better to the large-budget regime.
Period cap: Whether $p_{\max}=48$ binds is untested. Section 4.9 shows that re-scoring the archive under a larger cap changes nothing, but that follows from no compiled policy having requested a longer period, so it cannot rule out that a loop run with a larger cap would find one.

### 5.2. Future Work

Several directions remain for future research. The most immediate is to re-run the agentic loop natively on signals from other domains and sampling rates, rather than transferring policies compiled on one series as in Section 4.6, so that generality of the procedure is established alongside generality of the artefacts. Section 4.10 tests one chronological split on this series; repeating it at other cut points, on the further channels, and across models would establish how the contraction it measures depends on the split itself. Online adaptation mechanisms that allow policies to adjust to changing environments over time are a further direction. The optimiser comparison of Section 4.7 could be extended along two axes: to grammar-aware search operators that exploit the rule structure directly, and to surrogate models that remain tractable at the large budgets where the classical optimisers overtake the loop. In addition, richer policy grammars that support more expressive decision rules while maintaining low computational overhead may further improve adaptive sampling performance. Re-running the loop under a larger period cap would settle whether $p_{\max}=48$ binds, and quantify the trade-off against Proposition 1, which weakens quadratically in $p_{\max}$. Running the loop with an open-weight model would establish whether the procedure requires a frontier model at all; this is a different question from the cross-model comparison of Section 4.5, which we deliberately do not read as a ranking. Finally, while the memory footprint is now measured from a cross-compiled build (Section 4.8), deploying the compiled policies on real low-power platforms would allow execution time and energy savings to be measured directly rather than bounded by an operation count.

## 6. Conclusions

We investigated using a large language model as an offline policy compiler for energy-constrained sensing, casting generation as an agentic loop in which a generator proposes policies, a deterministic evaluator scores them exactly on the recorded trace, and an optimizer refines them from that feedback. Across four models and five seeds on the Beijing PM_2.5_ series, the loop improves on both its zero-shot output and a budget-matched random search over the identical grammar (pooled Wilcoxon $p<10^{-5}$ in both cases), and leads NSGA-II and Bayesian optimisation at matched evaluation budget, while the deployed policies remain compact, interpretable, and free of any on-device model. Under a chronological split of the same series the margin over unguided search survives on a held-out year in every run, contracting by roughly 42%.

Our analytical characterisation clarifies why. Reconstruction error under piecewise-linear interpolation is bounded by the signal’s second-order curvature (Lemma 1, Proposition 1), whereas the grammar exposes only first-order features; a causal, first-order policy therefore has limited access to the quantity that drives error, and can be expected to track rather than beat the frontier.

The contribution is thus a training-free but statistics-informed procedure: no weights are fitted and the raw series is never shown to the model, though eight summary scalars are. It yields deployable, interpretable policies which improve over a budget-matched random search over the same grammar, combining a compact, readable rule artefact with a measured gain over its references. The gain is budget-dependent, narrowing at the most aggressive subsampling, where the signal’s second-order curvature and strong short-lag autocorrelation leave little room for any first-order policy to separate from the frontier; this limit is what the analytical characterisation makes precise. Future work will broaden the evidence base along the axes identified in Section 5.1: running the loop natively on signals from other domains rather than transferring compiled policies, more seeds per model with reported dispersion, and curvature-aware features or richer reconstruction operators, followed by validation on live battery-powered hardware.

## Figures and Tables

**Table 1:** Positioning of the present approach relative to prior sampling and on-device intelligence strategies. “On-device cost” refers to the memory and computation required by the deployed artefact; “pre-deployment fitting” to any dataset-specific training or tuning needed before deployment.

Approach	Adaptivity	On-Device Cost	Interpretable	Pre-Deployment Fitting
Fixed-rate (periodic) [4]	None	Minimal	Yes	None
Delta-triggered rule [7]	Threshold	Minimal	Yes	Manual threshold
Event-triggered control [20,21]	Signal-driven	Low	Partial	Manual/tuned
Prediction-based [6]	Signal-driven	Low–mod.	Partial	Manual/tuned
TinyML models [8,9]	Learned	Mod.–high	No	Training req.
LLM heuristic search [15,16,35]	Signal-driven	Offline only	Yes	None
Black-box optimisers [18,19]	Signal-driven	Offline only	Yes	Per-signal search
This work	Signal-driven	Minimal	Yes	No training; summary stats only

**Table 2:** Ablation on the number of feedback rounds *R* with the number of proposals per round held at K=12. Each row is the state of the same twenty runs (four models × five seeds) truncated after round *R*; because the prompt at round *r* depends only on rounds <r, a truncated trajectory is exactly the outcome of a run configured with that *R*. Hypervolume and RMSE are means over the twenty runs, ± one standard deviation across runs. ΔHV is the gain over the preceding row. Cost is the mean cumulative API spend per run at provider list prices as of 11 August 2026 (standard non-batch tier), using the upper of the two output-token reconstructions of Section 4.8; the lower reconstruction gives $0.40 rather than $0.73 at R=8. The budget-matched random search, which evaluates 300  policies at every row, attains a mean hypervolume of 79.86 over the same twenty runs, stated as a mean to match the hypervolume column; its median is 80.25. An en dash indicates that ΔHV is not applicable for R=0.

*R*	Calls	Policies	Hypervolume	ΔHV	RMSE @ d≤0.10	RMSE @ d≤0.20	Cost (USD)
0	1	12	76.98 ± 2.92	–	42.07	27.45	0.069
1	2	24	80.51 ± 0.88	+3.53	41.93	26.28	0.148
2	3	36	80.97 ± 0.98	+0.46	40.58	25.49	0.230
3	4	48	81.21 ± 0.99	+0.25	40.23	24.79	0.306
4	5	60	81.38 ± 0.94	+0.17	39.83	24.72	0.390
5	6	72	81.60 ± 0.97	+0.22	39.73	24.28	0.471
6	7	84	81.78 ± 0.93	+0.18	39.70	23.95	0.561
7	8	96	81.85 ± 0.95	+0.07	39.70	23.91	0.646
8	9	108	81.92 ± 0.98	+0.07	39.37	23.58	0.731

**Table 3:** Behaviour at a matched evaluation budget. Each arm spends (R+1)K=108 policy evaluations and differs only in how they are scheduled. Two models (Claude Fable 5, ChatGPT 5.5) × five seeds per arm, paired by seed, all generated in August 2026. Hypervolume is the mean over the ten runs ± one standard deviation; larger is better. Calls is the number of API requests a run issues. Realised evaluations exceed 108 where ChatGPT 5.5 returned more policies than requested. Bold marks the best value in each of the three result columns, that is the highest hypervolume and the lowest RMSE.

*R*	*K*	Calls	Evaluations	Hypervolume	RMSE @ d≤0.10	RMSE @ d≤0.20
8	12	9	108.0	82.30 ± 0.54	**35.43**	22.60
5	18	6	108.5	**82.87** ± 0.38	36.34	**22.15**
3	27	4	108.8	**82.87** ± 0.92	36.76	22.48

**Table 4:** Final looped-front hypervolume per model (median across five seeds, with a bootstrap 95% confidence interval), compared with each model’s zero-shot front and the budget-matched random search (median hypervolume 80.25). Larger is better. Per-model *p*-values are paired Wilcoxon signed-rank across seeds; at n=5 their attainable minimum is 0.0625, so the pooled test is reported as an aggregate check rather than as a model-level pairwise claim.

Model	Loop HV	95% CI	Zero-Shot HV	*p* vs. 0-Shot	*p* vs. Random
Claude Fable 5	82.84	[82.41, 83.74]	79.28	0.0625	0.0625
ChatGPT 5.5	82.39	[82.07, 82.59]	79.05	0.0625	0.0625
Claude Opus 4.8	81.44	[80.94, 82.55]	77.09	0.0625	0.0625
ChatGPT 5.4	80.76	[80.40, 80.96]	74.44	0.0625	0.125

**Table 5:** Best reconstruction RMSE attained within a duty-cycle budget (lower is better). The four model columns and the random-search column are the mean across five seeds, with a bootstrap 95% confidence interval beneath. The three classical families are deterministic—a fixed period or a fixed threshold yields one trajectory—and therefore carry no interval. Bold marks looped means that improve on the best non-LLM alternative in that row by a margin exceeding their confidence interval; Fable 5’s nominal 0.02 advantage at d≤0.10 lies far inside both intervals and is therefore not marked. Em-dashes mark budgets the prediction-based family cannot reach: its sparsest configuration (τ=40) attains a duty cycle of 0.225.

Budget	Fable 5	Opus 4.8	ChatGPT 5.4	ChatGPT 5.5	Random	Fixed	Delta	Pred.
d≤0.10	35.29	40.66	44.38	37.15	35.31	37.29	37.95	—
	[34.49, 36.08]	[38.14, 43.17]	[42.66, 46.11]	[35.50, 38.80]	[34.51, 36.11]			
d≤0.15	**26.84**	29.22	33.26	**27.42**	28.47	29.45	35.64	—
	[26.37, 27.30]	[27.62, 30.82]	[32.62, 33.91]	[26.74, 28.10]	[27.69, 29.25]			
d≤0.20	**21.79**	24.88	25.52	**22.13**	25.09	24.99	35.04	—
	[21.14, 22.44]	[23.20, 26.56]	[24.57, 26.47]	[21.66, 22.60]	[23.86, 26.31]			
d≤0.30	**15.98**	**17.27**	**17.69**	**16.45**	20.57	19.82	30.74	25.03
	[15.24, 16.73]	[16.59, 17.95]	[16.31, 19.08]	[15.87, 17.04]	[19.56, 21.58]			
d≤0.50	**9.75**	**9.97**	**10.39**	**9.93**	11.74	10.73	26.99	15.12
	[9.42, 10.08]	[9.72, 10.22]	[10.17, 10.61]	[9.58, 10.28]	[9.76, 13.72]			

**Table 6:** Generalisation of PM_2.5_-compiled policies to four further channels, applied unchanged. Hypervolume reference is $\left(1.0,\sigma_{x}\right)$ per channel; larger is better. “Room” is $\sigma_{x}$ minus the fixed-rate hypervolume, i.e., the margin available to any adaptive policy; “captured” is the transferred policies’ gain over fixed-rate as a fraction of it. Random search is the median over five seeds at 300 policies. Channels are ordered by maximum discrete curvature $M_{⋆}$. Bold marks the channels on which the transferred policies exceed random search by more than one hypervolume unit.

Channel	$M_{⋆}$	Transferred	Random	Fixed-Rate	Room	Captured
PM_2.5_ (particulates)	863	**75.90**	71.38	71.02	19.93	25%
Wind speed (Iws)	588	**42.44**	37.53	37.08	12.93	41%
Dew point (DEWP)	24	13.22	13.14	13.11	1.33	8%
Temperature (TEMP)	15	10.95	10.87	10.81	1.39	10%
Pressure (PRES)	10	9.58	9.56	9.53	0.74	6%

**Table 7:** Median Pareto hypervolume over five seeds for the agentic loop and three black-box optimisers over the identical grammar, at matched and increasing policy-evaluation budgets. Larger is better. The loop is reported at the 108 evaluations it spends. ParEGO is reported at the matched budget only, since Gaussian-process fitting scales cubically in the number of observations. Bold marks the highest hypervolume at the matched budget of 108 evaluations and the highest attained at any budget. An em dash indicates that the method was not run at that budget.

Optimiser	108	300	1000	3000
Agentic LLM loop (this work)	**82.11**	—	—	—
ParEGO (Bayesian optimisation)	77.77	—	—	—
NSGA-II	77.40	78.23	81.28	**84.11**
Random search	73.99	80.25	81.05	82.18

**Table 8:** Configuration and cost of the NSGA-II baseline against the agentic loop. Population, operators and archive policy are those that produced Table 7. The archive is unbounded, so its size equals the evaluation budget; “front” is the size of its non-dominated subset at termination. Hypervolume is the median over five seeds. Wall-clock is the median over five seeds of the same deterministic evaluator on one CPU core, at 3.29 ms per policy evaluation; for the loop it is evaluator time only, since API latency was not instrumented (Section 4.8). For the agentic loop, “gens” is the number of feedback rounds *R*, and population and front are not defined, since the loop maintains a non-dominated archive rather than a generational population; an em dash marks those two entries. Bold marks the highest hypervolume in the table.

Method	Evals	Pop.	Gens	Archive	Front	HV	Wall-Clock
NSGA-II	108	24	4	108	42	77.40	0.5 s
NSGA-II	300	24	12	300	103	78.23	4.1 s
NSGA-II	1000	24	41	1000	329	81.28	16.1 s
NSGA-II	3000	24	124	3000	777	**84.11**	49.0 s
Agentic loop (this work)	108	—	8	108	—	82.11	0.4 s

**Table 9:** Deployed-artefact cost over all 2161 compiled policies. Upper panel: the stated encoding. Flash is the encoded policy under four-byte alignment; RAM is the causal feature state; operations are floating-point operations per decision point. The seven-byte packed encoding gives a worst case of 79 bytes. Lower panel: section sizes measured with arm-none-eabi-size after compiling the same policies as static C data with GCC 13.2 at -Os, at object level so that startup code and the C library are excluded. The interpreter is independent of the policy and is charged once. Measured .rodata matches the encoding exactly; measured .bss falls below it for the reason given in the text.

Quantity	Median	Worst Case
Stated encoding		
Rules per policy	5	11
Flash (bytes)	44	92
RAM (bytes)	20	28
Operations per decision	17	28
Measured, Cortex-M4F/Cortex-M0+		
Policy table .rodata (bytes)	44	92
Interpreter .text (bytes)	296/320	296/320
Feature state .bss (bytes)	16	16
Total flash (bytes)	340/364	388/412

**Table 10:** Offline compilation cost per run, by model, reconstructed from the recorded prompts and policies without further API calls. Input tokens are exact; output tokens are a reconstruction reported as a lower and upper bound, and the cost range follows from it. Values are means across the five seeds. Costs are at provider list prices as of 11 August 2026 (standard non-batch tier, no prompt caching), on the same basis as Table 2.

Model	Calls	Input Tokens	Output Tokens	Cost (USD)
Claude Fable 5	9	9023	9488–18,683	0.56–1.02
Claude Opus 4.8	9	8556	9867–19,598	0.29–0.53
ChatGPT 5.4	9	9376	14,492–28,347	0.24–0.45
ChatGPT 5.5	9	9533	14,971–29,020	0.50–0.92
Study total (20 runs)	180	182,445	244,094–478,244	7.96–14.62

**Table 11:** Sensitivity of the pooled results to the two constants fixed a priori in Section 3.3. All 2161 policies compiled across the twenty runs were re-scored under each combination by the same deterministic evaluator; no further API calls were issued. Upper panel: pooled Pareto hypervolume (larger is better). Lower panel: best RMSE within a 0.20 duty-cycle budget (lower is better). The published setting is $\left(\alpha ,p_{\max}\right)=\left(0.20,48\right)$, marked ^†^. The $p_{\max}\geq 48$ columns are identical because the longest period requested by any compiled policy is 48; see the text.

	$p_{\max}$				
α	**12**	**24**	**48**	**96**	**168**
Pareto hypervolume					
0.05	79.96	81.69	82.18	82.18	82.18
0.10	79.92	81.76	82.21	82.21	82.21
0.20	79.65	81.57	82.11 ^†^	82.11	82.11
0.40	79.31	80.93	81.62	81.62	81.62
0.80	77.37	79.15	79.80	79.80	79.80
Best RMSE at d≤0.20					
0.05	22.64	22.88	22.88	22.88	22.88
0.10	22.62	22.64	22.65	22.65	22.65
0.20	22.68	22.86	22.86 ^†^	22.86	22.86
0.40	22.82	23.09	23.09	23.09	23.09
0.80	25.48	26.86	26.86	26.86	26.86

**Table 12:** Chronological split. Policies are compiled on 2010–2013 (35,064 h) and scored on the held-out 2014 segment (8760 h). The training segment supplies the eight prompt scalars and every score the loop is shown; the random-search control and the classical families are recompiled on the same training segment. Both the loop and the control have their fronts selected on the training segment, so neither uses the held-out year to choose. Hypervolume is the mean over the six runs ± one standard deviation, against the fixed reference (1.0,100) used throughout; larger is better. Levels are not comparable between the two columns, since every method scores higher on the held-out segment; the margin is. An em dash indicates that the margin is not applicable to the agentic loop itself.

Method	HV, 2010–2013	HV, 2014	Margin (in/out)
Agentic loop	82.02 ± 0.42	82.85 ± 0.40	—
Random search	79.58 ± 0.98	81.44 ± 1.00	+2.44/+1.42
Classical families	80.70	81.97	+1.32/+0.88

**Table 13:** Best reconstruction RMSE within a duty-cycle budget on the held-out 2014 segment (lower is better). Every column is selected on the training segment and scored on the held-out one. Means across the six runs. The loop leads only at the two loosest budgets; at d≤0.20 and below the random-search control is at least as good, which is the same budget dependence reported in Table 5 in sample, more pronounced out of it. Bold marks the lowest RMSE among the three held-out columns in each row.

Budget	Loop, 2010–2013	Loop, 2014	Random, 2014	Classical, 2014
d≤0.10	37.75	35.06	**31.81**	34.01
d≤0.15	27.87	25.33	**24.97**	27.13
d≤0.20	22.63	20.55	**20.26**	23.28
d≤0.30	17.38	**16.33**	18.47	17.44
d≤0.50	10.07	**9.64**	10.66	9.84

## Data Availability

The Beijing PM_2.5_ dataset analysed in this study is publicly available as described in Section 3.4. The code used for policy evaluation, baseline generation, random search, Pareto-front construction, statistical analysis, the NSGA-II and ParEGO baselines, the cross-channel replay, the sensitivity analysis over the smoothing factor and period cap, the ablation on the number of feedback rounds, the matched-budget (R,K) arms, the NSGA-II convergence and cost analysis, the cross-compiled footprint measurement and the C it generates, the chronological split and its analysis, and figure generation, together with the complete archive of all generated policies and their recorded scores—the 2161 policies of the twenty July runs, the 3253 policies of the thirty August matched-budget runs, and the 612 policies of the six chronological-split runs, each with its scores on both segments—is available at: https://github.com/sarabehnamian/agentic-llm-sampling-policies (accessed on 26 August 2026).

## Appendix A. Generated Policies

This appendix gives the compiled artefacts themselves, since the policy—not the loop—is what a deployment would carry.

Table A1 lists, for each duty-cycle budget of Table 5, the single best-scoring policy compiled anywhere in the study, exactly as the model emitted it and as the evaluator executed it. Rules are tested in order and the first match sets the next sampling period; if none matches, the default period applies. Every entry is a complete artefact: no state beyond the features of Section 3.3 is retained, and nothing else is deployed.

These are best-of-study policies, not per-budget means, so their RMSE values sit below the corresponding entries of Table 5, which average five seeds per model. All five happen to come from Claude Fable 5, the model with the highest median hypervolume (Section 4.5); we note this without reading an ordering into it, for the reasons given there.

**Table A1:** Best policy compiled in the study within each duty-cycle budget, as emitted. Rules are evaluated in order; the first match sets the period, otherwise the default applies. Duty and RMSE are the exact scores from the trace-driven evaluator. These are single best-of-study artefacts, so they score below the five-seed means of Table 5.

Budget	Duty	RMSE	Rule Set
d≤0.10	0.0942	34.23	ewm_var >2500→8
			ewm_var >400→16
			default →32
d≤0.15	0.1496	26.15	abs_delta >55→3
			ewm_var >1500→6
			ewm_var <70→28
			ewm_var <250→20
			default →14
d≤0.20	0.1984	20.99	ewm_var >1500→4
			ewm_var >600→7
			ewm_var <80→22
			ewm_var <250→14
			default →10
d≤0.30	0.2937	15.24	abs_delta >35→2
			ewm_var >1000→3
			ewm_var >250→6
			ewm_var <40→24
			default →10
d≤0.50	0.4915	9.29	abs_delta >25→1
			ewm_var >600→2
			ewm_var >150→3
			ewm_var >40→6
			ewm_var <10→18
			default →12

The recurring structure asserted in Section 4 is visible in every entry and can be stated with a number. Across all 2161 compiled policies, comprising 10,338 rules, abs_delta accounts for 40.1% of rules and ewm_var for 35.8%, with slope at 15.0% and rel_delta at 9.1%; the median policy carries five rules and the largest eleven. The compiled policies therefore concentrate on the two features that most directly signal local volatility, sampling densely when the last observed change is large or the exponentially weighted variance is high, and sleeping when it is low.

One feature is entirely absent: time_since appears in none of the 10,338 rules. This complements the ablation of Section 4.7, where including the constant feature was shown to strengthen the random-search control by injecting fixed-rate policies into its pool. The generator, given the same grammar, never used it. The two observations agree: the feature is useful only as an accidental route to constant-period sampling, and guided generation had no need of that route.

The five policies above are illustrative. The complete archive of all 2161 generated policies, each with the exact duty cycle and RMSE recorded by the evaluator, is released as a single JSON file in the repository named in the data availability statement, so that every number in this paper can be regenerated from it without issuing an API call.

## Appendix B. Prompt Templates

The loop issues two prompts. Both are given here in full, since the natural-language specification is the input the compiler view is organised around, and paraphrasing it would leave the method underdetermined. Both are assembled by string interpolation from the signal summary of Section 3.4 and the feedback digest, so the text below is what the model actually received, modulo those substitutions.

Both prompts embed a shared grammar block, reproduced once here and referenced as grammar in the two templates that follow.

* *

Policy format (JSON). Rules are checked in order; first match wins.

Available O(1) on-device features (computed only from ALREADY-SAMPLED values):

  abs_delta   |last sample - previous sample|

  rel_delta   abs_delta / (|previous|+1)

  ewm_var     exp. weighted variance of samples (alpha=0.2)

  slope       change per step between last two samples

  time_since  steps since last sample (always 0 at decision time)

Each rule: {"if": {"feature": <f>, "op": ">"|"<", "thr": <number>},

            "period": <int 1..48>}

Plus a top-level "default_period" (int 1..48) used when no rule~matches.

* *

Example:

{"name":"example","rules":[

  {"if":{"feature":"abs_delta","op":">","thr":30},"period":1},

  {"if":{"feature":"ewm_var","op":"<","thr":100},"period":24}],

 "default_period":6}

### Appendix B.1. Generator Prompt (Round 0)

The base template. {summary} is the eight-scalar JSON object of Section 3.4; {k} is K=12.

* *

You  are designing sampling policies for a battery-powered air-quality sensor.

Each time it samples, the~device decides how long to wait (period 1..48 steps) until

the next sample. Goal: minimize samples taken (energy) while keeping linear-interpolation

reconstruction RMSE~low.

* *

Signal statistics: {summary}

* *

<GRAMMAR>

* *

Generate {k} diverse candidate policies as a JSON array covering different points of the

energy/fidelity tradeoff (some aggressive/very few samples, some conservative). Use the

statistics to pick sensible thresholds. Respond ONLY with the JSON array.

### Appendix B.2. Prompt Variants

Four variants probe sensitivity to wording; the run seed selects one, and it is used for every round of that run. Variant 1 is the base template above. The remaining three modify it as follows.

* *

Variant 2 -- appended to the base template:

* *

  Prioritise covering the LOW-POWER regime (duty<=0.2) well.

* *

Variant 3 -- appended to the base template:

* *

  Think step by step about the curvature/excursions implied by the statistics,

  then output only the JSON~array.

* *

Variant 4 -- substitution within the base template. The~phrase

  "diverse candidate policies"

is replaced by

  "policies that each target a DIFFERENT duty-cycle band

   (0.03-0.05, 0.05-0.1, 0.1-0.2, 0.2-0.35, 0.35-0.6)"

### Appendix B.3. Optimizer Prompt (Rounds 1–8)

The feedback template. Three blocks are filled from the evaluator’s measured scores: the current non-dominated front (up to 20 entries), the duty-cycle budgets at which a classical baseline still attains lower error, and a seed-shuffled sample of six underperforming policies from the run so far. The seed enters only through that sample, which is what makes repeated runs diverge.

* *

IMPROVE THE POLICIES. This is an optimization~round.

* *

Signal statistics: {summary}

* *

<GRAMMAR>

* *

Current best-known (Pareto) policies and their MEASURED scores:

  - <name>: duty=<d>, rmse=<r>          [up to 20 lines]

* *

Budgets where a classical baseline still beats your policies (close these gaps):

  - budget d<=<b>: best classical rmse=<c>, your best rmse=<l>

* *

Some of your policies that underperformed (fix or replace them):

  - <name>: duty=<d>, rmse=<r>          [6 lines, seed-shuffled]

* *

Produce {k} NEW improved policies as a JSON array. Focus on the budgets where you

are currently behind: reduce RMSE without exceeding that duty budget. You may keep

and refine good structures from the front above. Respond ONLY with the JSON array.

* *

Two properties of these templates bear on claims made elsewhere. First, the only signal-derived content in either prompt is the eight-scalar summary; no raw sample is transmitted, which is the precise sense in which the method is statistics-informed rather than trained (Section 3.5). Second, every score shown to the optimizer is a measured output of the deterministic evaluator, never a model estimate, which is what makes the feedback trustworthy and the comparison against random search unambiguous (Section 2.5).
